# Activity map of a cortico-cerebellar loop underlying motor planning

**DOI:** 10.1038/s41593-023-01453-x

**Published:** 2023-10-09

**Authors:** Jia Zhu, Hana Hasanbegović, Liu D. Liu, Zhenyu Gao, Nuo Li

**Affiliations:** 1 Department of Neuroscience, Baylor College of Medicine, Houston, TX USA; 2 Department of Neuroscience, Erasmus MC, Rotterdam, the Netherlands

**Keywords:** Neural circuits, Cognitive control, Cerebellum

## Abstract

The neocortex and cerebellum interact to mediate cognitive functions. It remains unknown how the two structures organize into functional networks to mediate specific behaviors. Here we delineate activity supporting motor planning in relation to the mesoscale cortico-cerebellar connectome. In mice planning directional licking based on short-term memory, preparatory activity instructing future movement depends on the anterior lateral motor cortex (ALM) and the cerebellum. Transneuronal tracing revealed divergent and largely open-loop connectivity between the ALM and distributed regions of the cerebellum. A cerebellum-wide survey of neuronal activity revealed enriched preparatory activity in hotspot regions with conjunctive input–output connectivity to the ALM. Perturbation experiments show that the conjunction regions were required for maintaining preparatory activity and correct subsequent movement. Other cerebellar regions contributed little to motor planning despite input or output connectivity to the ALM. These results identify a functional cortico-cerebellar loop and suggest the cerebellar cortex selectively establishes reciprocal cortico-cerebellar communications to orchestrate motor planning.

## Abstract (web summary)

The neocortex and cerebellum interact during cognitive functions. The authors assembled an activity map of the cerebellum in relation to its connectome with frontal cortex, which uncovered how the two areas form networks to orchestrate motor planning.

## Main

The neocortex and the cerebellum are thought to interact during motor and nonmotor functions^1,2^. The two brain regions are reciprocally linked through the pons and thalamus to form cortico-cerebellar loops^3,4^. Recent works suggest that cortico-cerebellar loops are crucial for coordinating neocortical and cerebellar activities underlying specific behaviors^5–12^. However, because the connections between the neocortex and the cerebellum go through multiple relay brain areas with complex topography^8,13–15^, it remains poorly understood how the neocortex and the cerebellum organize into functional networks to drive behavior.

During volitional movements, movements are preceded by a planning phase in which neural activity evolves into a state of readiness for prepared actions^16,17^. Preparatory activity is postulated to emerge from distributed processes that involve cortico-cerebellar loops^6,11,12^. Preparatory activity is observed in the frontal cortex^18–23^, pons^24^, thalamus^25,26^ and the cerebellum^6,11,12,27,28^. Recent evidence shows that preparatory activity in the frontal cortex depends on the thalamus^26,29^ and cerebellar nuclei^11,12^.

A frontal cortical region in the mouse, the ALM, is necessary for planning and initiation of directional licking^30–32^. Parts of the cerebellum, particularly Crus 1 and 2, have also been implicated in the control of orofacial movements^28,33,34^. The ALM projects to the cerebellum via the basal pontine nuclei, and the cerebellar nuclei outputs target the ALM-projecting thalamus^11,13,15^. Perturbations of the fastigial nucleus abolish ALM preparatory activity and bias the direction of future licking^11^. Beyond the involvement of these key network nodes, the critical missing link is the cerebellar cortex, which connects neocortical inputs to the cerebellar nuclei. Distribution of preparatory activity in the cerebellar cortex is not well understood. It is thus unknown how the ALM and the cerebellum form functional networks during motor planning of orofacial movements, and whether preparatory activity is orchestrated by information flow from the neocortex to cerebellum or from the cerebellum to neocortex, or both.

We delineated activity in the cerebellar cortex supporting motor planning of directional licking in relation to the mesoscale cortico-cerebellar connectome. Contrary to previously proposed closed-loop architecture between specific neocortical and cerebellar regions^3^, transneuronal tracing revealed highly divergent and largely open-loop connectivity between the ALM and large swaths of the cerebellar cortex. To understand the functional consequence of this connectivity, we assembled an activity map of the cerebellar cortex during a delayed response task in which mice used short-term memory to plan directional licking. Notably, preparatory activity instructing future lick direction was enriched only in patches of cerebellar regions with a conjunction of input–output connectivity to the ALM. Perturbation experiments show that the conjunction regions were required for maintaining preparatory activity. Interestingly, cerebellar regions with only input or output connectivity to the ALM contributed little to preparatory activity and the motor planning behavior. These results identify a cortico-cerebellar loop for motor planning of orofacial movements, and they show that preparatory activity is orchestrated by reciprocal communication between the ALM and the cerebellum.

## Results

### Divergent and convergent connectivity of cerebellar cortex with the ALM

**Fig. 1:**
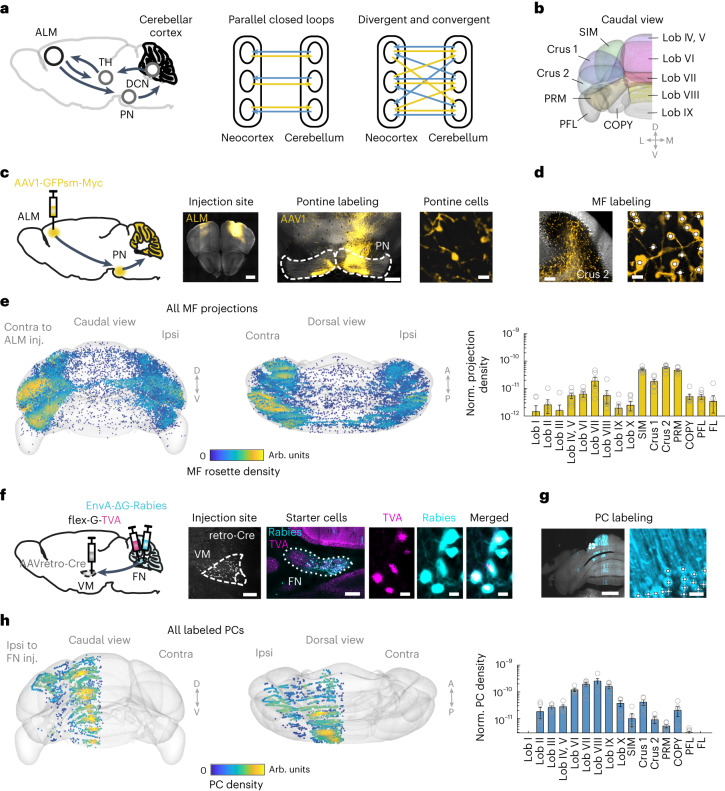
Divergent and convergent connectivity of the ALM with cerebellar cortex. **a**, ALM cortico-cerebellar loop (left) and possible schemes of connectivity (right). TH, thalamus; DCN, deep cerebellar nuclei; PN, pontine nuclei. Yellow arrows indicate descending cortico-cerebellar pathways. Blue arrows indicate ascending cerebello-cortical pathways. **b**, Cerebellar cortex from the Allen Reference Brain with lobule annotations. **c**, Left, anterograde AAV1 transneuronal tracing of ALM inputs to cerebellar cortex. Right, ALM injection site and labeling in the basal pontine nuclei. Fluorescence outside the pons is from labeled axons. Magnified image shows labeled pontine cells. This experiment was repeated on seven mice with similar results. Histology images from one mouse are displayed. Scale bars, 500 µm, 200 µm and 10 µm. **d**, Left, example image of labeled mossy fibers in the cerebellar cortex from one mouse. Right, magnified image of mossy fiber terminals. Dots indicate annotated mossy fiber terminals. Scale bars, 100 µm and 10 µm. MF, mossy fiber. **e**, Left, ALM inputs to the cerebellum. Dots show annotated mossy fiber terminals from all injection cases (*n* = 7 mice). Color reflects the kernel density estimation of inputs (Methods). Mossy fiber annotations are aligned into the Allen Mouse CCF. Right, normalized mossy fiber density in each lobule. Both hemispheres are combined (Methods). Mean ± s.e.m. across mice. Circles indicate individual mice. SIM, simplex lobule; PRM, paramedian lobule; COPY, copula pyramidis; PFL, paraflocculus; FL, flocculus. **f**, Left, retrograde rabies tracing from ventromedial thalamic nucleus (VM) and fastigial nucleus (FN) labels upstream Purkinje cells in the cerebellar cortex. Right, VM injection site and labeled starter cells in the fastigial nucleus. This experiment was repeated in five mice with similar results. Histology images from one mouse are displayed. Scale bars, 500 µm, 200 µm and 10 µm. **g**, Left, example image of labeled Purkinje cells (cyan) from one mouse. Right, magnified image of labeled Purkinje cells in boxed area from the left image. Dots indicate annotated Purkinje cell somas. Scale bars, 100 µm and 10 µm. This experiment was repeated in five mice with similar results. **h**, Left, Purkinje cells targeting ALM-projecting thalamus in caudal (left) and dorsal (right) view. Dots show annotated Purkinje cells from all injection cases (*n* = 5 mice). Color reflects the kernel density estimation of Purkinje cells. PC, Purkinje cell. Right, normalized Purkinje cell density in each lobule. Mean ± s.e.m. across mice. Circles indicate individual mice. Source data

Previous anatomical tracings using sparse transneuronal tracing suggest a closed-loop architecture between the neocortex and cerebellum, where specific regions of the cerebellum receive inputs from specific areas of neocortex and in turn project back to the same neocortical areas^3^. On the other hand, recent transneuronal tracings reveal a high degree of divergence and convergence between the neocortex and cerebellum with complex topography^8,13–15^ (Fig. 1a). We examined which cerebellar regions formed anatomical loops with the ALM by mapping their reciprocal connectivity.

**Extended Data Fig. 1:**
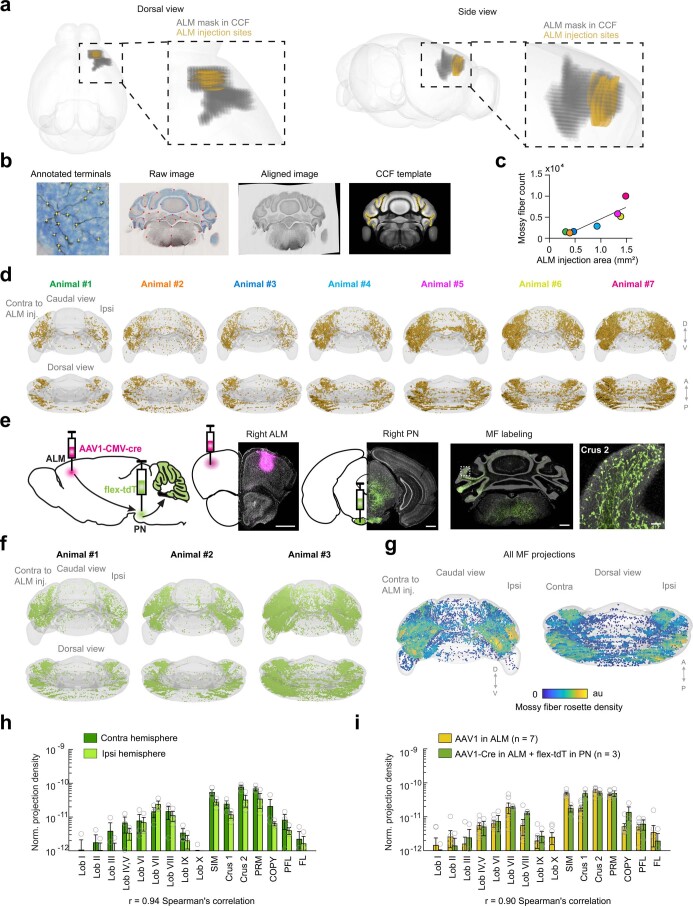
Anterograde tracing of ALM inputs to cerebellar cortex. **a**. Quantifications of injections with respect to ALM border in CCF. ALM border defined in^30^ and^41^. Injections were restricted in ALM. **b**. Registration of ALM inputs. Mossy fiber terminals labeled by AAV1 injection in ALM are manually annotated in coronal sections (yellow crosses). Consecutive coronal sections covering the cerebellum are aligned into the Allen Mouse Common Coordinate Framework (CCF). The image shows an example coronal section before and after alignment to the corresponding section of the anatomical template in CCF. Red points indicate landmark points for b-spline transformation. Annotated mossy fiber terminals in the coronal section are shown in CCF. **c**.The number of labeled mossy fiber terminals is correlated with viral infection area in ALM (n = 7 mice). Color codes for individual mice as in **d**. **d**.ALM inputs in individual injection cases in caudal and dorsal view. Dots show annotated mossy fiber terminals. The pattern is similar across injection cases. **e**. Control experiment to specifically label mossy fiber projections arising from ALM-recipient pontine nuclei. From left to right, injection schematics; images of injection sites in right ALM and right pons; images of labeled mossy fiber terminals in cerebellum. Fluorescence outside of the pons and the cerebellar cortex are from labeled axons. Histology images from one mouse are displayed. Injections were repeated in 3 mice with similar results. Scale bars, 500 µm, 500 µm, 500 µm and 100 µm. **f**. ALM inputs in individual injection cases from **e** in caudal and dorsal view. **g**. Summary of ALM inputs across all injection cases (n = 3 mice) in **f**. Same as Fig. 1e. Color codes for kernel density estimation of ALM inputs. **h**. Normalized ALM input density in contralateral and ipsilateral hemispheres in AAV1-cre injection experiment. Data from injection cases (n = 3 mice) in **f**. Open circles, individual mice. Error bars, s.e.m. across mice. **i**. Comparison of ALM input density labeled by direct AAV1 injections in ALM (Fig. 1e) and Cre-dependent expression of tdTomato in ALM-recipient pontine nuclei (panel **f**). Both hemispheres are combined to calculate mossy fiber density. Open circles, individual mice. Error bars, s.e.m. across mice. Source data

We mapped ALM inputs to the cerebellar cortex by localizing regions innervated by mossy fibers arising from the ALM-recipient pontine nuclei. To investigate this topography, we took advantage of the anterograde transsynaptic property of the adeno-associated virus serotype 1 (AAV1)^35^. AAV1 was injected into the ALM (Extended Data Fig. 1a), entering a subset of downstream pontine neurons (Fig. 1c). Labeled mossy fiber terminals could be easily distinguished as large boutons in the cerebellar cortex (also known as rosettes; Fig. 1d). We imaged coronal sections across the entire cerebellar cortex and annotated the mossy fiber terminals (Extended Data Fig. 1b and Methods). Coronal sections were aligned into the Allen Mouse Common Coordinate Framework (CCFv3; Methods)^36^. The topography of mossy fiber terminals was consistent across individual ALM injections, despite variability in infection rate (Extended Data Fig. 1c,d; *n* = 7 mice). We thus used the relative density of mossy fiber terminals as a proxy for the ALM input map in the cerebellar cortex (Methods).

**Extended Data Fig. 2:**
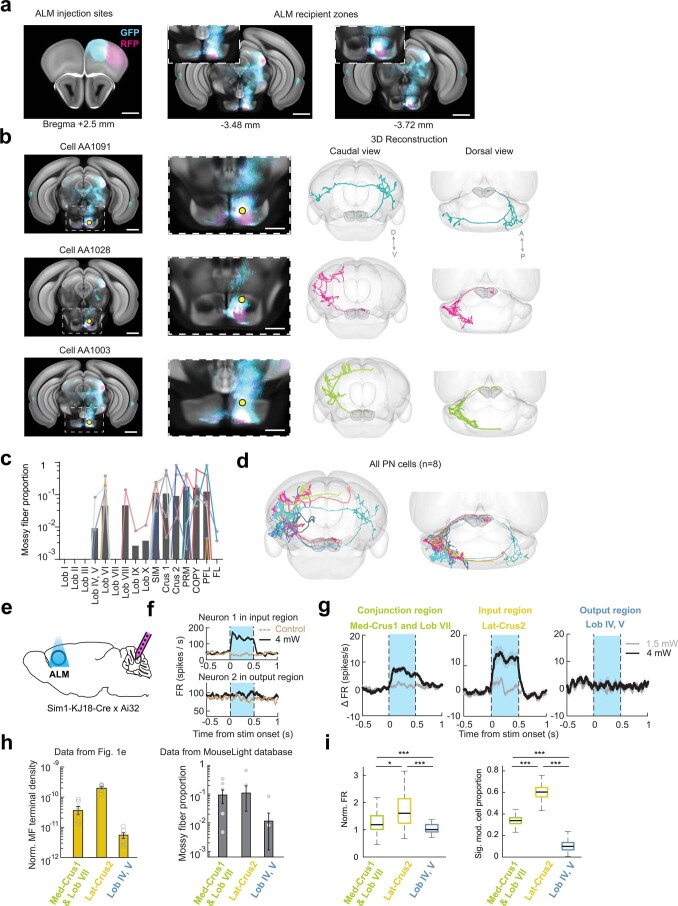
Single pontine neurons send divergent projections to cerebellar cortex. **a**. Two-colored anterograde tracer injections in the medial and lateral ALM to map ALM-recipient pons. Left, registered coronal section showing a medial (cyan) and lateral (magenta) ALM injection site in CCF. Scale bar, 1 mm. Right, labeling in the basal pontine nuclei. Inset, zoom in of pontine labeling. White shows overlap between medial and lateral ALM projection zones. Scale bar, 1 mm. **b**. Reconstruction of individual pontine neurons located in ALM-recipient pons from the MouseLight database (http://ml-neuronbrowser.janelia.org/). Three example neurons are shown. Left, registered coronal sections showing individual soma locations overlaid on ALM projections from **a**. Yellow dots, soma. Scale bar, 1 mm. Middle, zoom in of the basal pontine nuclei. Scale bar, 500 µm. Right, single neuron reconstructions in caudal (left) and dorsal view (right). Individual pontine neurons send divergent projections to cerebellar cortex. **c**. Proportion of mossy fibers in different lobules of the cerebellar cortex. Individual lines, proportion of mossy fibers in different lobules for individual pontine neurons (n = 8). Bar, mean across neurons. **d**. 3D Reconstruction of all pontine neurons located in ALM-recipient pons. Colors reflect individual neurons. Left, caudal view; right, dorsal view. **e**. Recording in cerebellar cortex and photostimulation in contralateral ALM. **f**. Firing rate of two example neurons in control and photostimulation trials. **g**. Population firing rate changes (mean ± s.e.m. across neurons) in conjunction regions (n = 202), input-dominant regions (n = 113) and output-dominant regions (n = 78). ∆Firing rate is the difference between photostimulation and control trials. Cyan, photostimulation epoch. **h**. Left, normalized mossy fiber terminal density in the recorded area. Data from Fig. 1e. Error bars, mean ±s.e.m. across mice (n = 7). Right, proportion of mossy fibers axons in the recorded area. Data from Extended Data Fig. 2c. Error bars, mean ±s.e.m. across neurons (n = 8). **i**. Left, photostimulation effect size on population firing rate. Normalization is calculated as the firing rate during photostimulation divided by firing rate in control trials during the same epoch. Box and whisker plot shows median, 25/75th percentiles and most extreme data points not considered as outliers. Kruskal-Wallis One-Way ANOVA Test, *H*(2) = 33.51, *P* = 5.28 × 10^−8^. Post-hoc pair-wise Mann Whitney U Test, conjunction vs. input-dominant, *P = 0.0137; conjunction vs. output-dominant, ***P = 6.3769 × 10^−6^, input-dominant vs. output-dominant, ***P = 6.6871 × 10^−8^. Right, proportion of neurons showing significant firing rate change during photostimulation. Data from 4 mW photostimulation condition. Box and whisker plot shows median, 25/75th percentiles and most extreme data points not considered as outliers (bootstrap, Methods). Conjunction vs. input region, *Χ*^2^(1) = 19.4713, *** *P* = 1.021 × 10^−5^, conjunction vs. output region, *Χ*^2^(1) = 24.7457, *** *P* = 6.541 × 10^−7^, input region vs. output region, *Χ*^2^(1) = 59.682, *** *P* = 1.115 × 10^−14^. Chi-squared test Conjunction region neurons (n = 202), input region neurons (n = 113) and output region neurons (n = 78) were sampled from 2 mice. Source data

The ALM provided divergent inputs to the cerebellar cortex, primarily targeting the hemispheres and posterior vermal regions (Fig. 1e). Each hemisphere of the ALM innervated both hemispheres of the cerebellar cortex, although the projections were stronger in the contralateral hemisphere (Fig. 1e and Extended Data Fig. 1h). AAV1 may infect neurons across multiple synapses, which may label other pathways that give rise to the diffused mossy fiber projections. To confirm this topography, we injected AAV1 encoding Cre recombinase into the ALM and a second Cre-dependent virus into the ipsilateral pontine nuclei to specifically label ALM-recipient neurons (*n* = 3 mice). The resulting input topography was similar (Extended Data Fig. 1e–g). Next, we analyzed axonal morphology of individual pontine neurons located in ALM-recipient pons from the MouseLight database (http://ml-neuronbrowser.janelia.org/)^37^. Individual pontine neurons innervated multiple regions of the cerebellar cortex with a pattern similar to the topography of labeled mossy fiber terminals (Extended Data Fig. 2a–d). Thus, the divergent input topography likely arises from diffused mossy fiber innervations of pontine neurons^38^. Finally, to verify that the density of mossy fiber terminals provided a proxy for functional connections, we photoactivated ALM layer 5b pyramidal-tract neurons innervating the pons while recording from distinct cerebellar regions (Sim1_KJ18-cre mice crossed with Ai32 mice; Methods). The light-evoked activity in different cerebellar regions was correlated with the density of mossy fiber terminals (Extended Data Fig. 2e–i). Altogether, these data show that a single spot in the frontal cortex sends divergent inputs to distributed regions of the cerebellar cortex.

**Extended Data Fig. 3:**
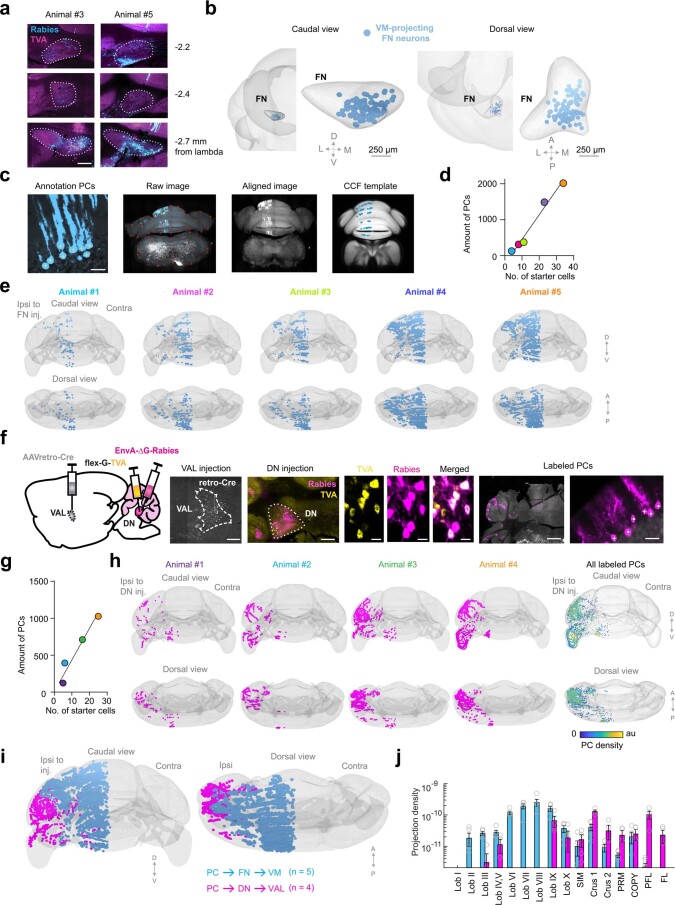
Retrograde tracing of Purkinje cells targeting ALM. **a**. Coronal sections from two example cases showing labeled neurons from retroAAV-Cre injection in ventromedial thalamic nucleus (VM) and Cre-dependent rabies viruses in the fastigial nucleus (Fig. 1f). Dashed line shows boundary of the fastigial nucleus. Purple, TVA positive neurons; blue, rabies positive neurons, that is starter cells. Scale bar, 250 µm. This experiment was repeated in 5 mice with similar results. **b**. Distribution of all starter cells in the fastigial nucleus. Data from all injection cases (n = 5). Each dot represents one neuron. VM-projecting fastigial neurons are concentrated in the caudal region of the fastigial nucleus. **c**. Registration of retrogradely labeled Purkinje cells (yellow crosses). Consecutive coronal sections covering the cerebellum are aligned into the Allen Mouse Common Coordinate Framework (CCF). The image shows an example coronal section before and after alignment to the corresponding section of the anatomical template in CCF. Red points indicate control points for b-spline transformation. Annotated Purkinje cells in the coronal section are shown in CCF. **d**. The number of labeled Purkinje cells is correlated with the number of starter cells in the fastigial nucleus (n = 5 mice). Color codes for individual animals as in **e**. **e**. Labeled Purkinje cells in individual injection cases in caudal and dorsal view. Dots show annotated Purkinje cells. The pattern is similar across injection cases. **f**. Retrograde tracing of Purkinje cells from ventral anterolateral thalamic nucleus (VAL) and dentate nucleus (DN). From left to right, injection schematics; images of injection sites in VAL and DN; images of labeled starter cells in DN, images of labeled Purkinje cells in cerebellar cortex. Histology images from one mouse are displayed. This experiment was repeated in 4 mice with similar results. Scale bars from left to right, 500 µm, 200 µm, 10 µm, 10 µm, 10 µm, 100 µm and 10 µm. **g**. The number of labeled Purkinje cells is correlated with number of starter cells in DN (n = 4 mice). Color codes for individual animals as in **h**. **h**. Labeled Purkinje cells in individual injection cases in caudal and dorsal view. Right, summary of Purkinje cells targeting VAL via DN. Color codes for kernel density estimation of Purkinje cells. **i**. Comparison of Purkinje cells targeting VM via FN (blue) and Purkinje cells targeting VAL via DN (magenta). **j**. Comparison of output Purkinje cell density from **i** in each lobule. Open circles, individual mice (labeling from FN n = 5 mice, DN n = 4 mice). Error bars, s.e.m. across mice. Source data

We next mapped cerebellar regions projecting back to the ALM. Cerebellar outputs are funneled through the cerebellar nuclei, which target the ALM through the thalamus (Fig. 1f). We focused on the fastigial nucleus due to its involvement in controlling orofacial movements: perturbation of the fastigial nucleus, and not the dentate nucleus, biases the direction of future licking^11^. We injected AAVretro^39^ encoding Cre recombinase in the ALM-projecting thalamus (around ventromedial nucleus (VM)), and AAV8 encoding flexed TVA and G proteins to label the VM-projecting fastigial neurons (Fig. 1f). VM-projecting neurons were localized to the caudal portion of the fastigial nucleus (Extended Data Fig. 3a,b)^15^. Subsequent unilateral injection of pseudotyped rabies viruses^40^ labeled their input Purkinje cells (Fig. 1f). Labeled Purkinje cells were comprehensively imaged and annotated across the cerebellar cortex (Fig. 1g, Extended Data Fig. 3c and Methods). Individual injections revealed similar patterns of labeling, despite variability in infection rate (Extended Data Fig. 3d,e; *n* = 5 mice). In contrast, retrograde rabies tracing from thalamic ventral-anterior-lateral nucleus and the dentate nucleus produced a distinct pattern of Purkinje cell labeling (Extended Data Fig. 3f–j; *n* = 4 mice). Thus, rabies-mediated transneuronal tracing labeled Purkinje cells in specific cerebello-cortical pathways.

Large swaths of the cerebellar cortex sent converging outputs to the ALM via the fastigial nucleus and thalamus. Labeled Purkinje cells were prevalent throughout the vermal regions, and also notably in parts of Crus 1/2 and the simplex lobule (Fig. 1h)^15^. Interestingly, the cerebellar regions providing outputs to the ALM were largely misaligned with the cerebellar regions receiving ALM inputs. Nevertheless, patches of cerebellar regions exhibited a conjunction of input and output labeling, covering parts of Crus 1/2, simplex lobule and posterior vermal regions around lobule VII. These regions thus link ALM inputs with cerebellar outputs back to the ALM, closing a cortico-cerebellar loop at the level of anatomy.

Thus, contrary to the notion of parallel cortico-cerebellar closed loops, we found highly divergent and largely open-loop connectivity between the ALM and the cerebellum, and patches of cerebellar regions have a conjunction of input–output connectivity to the ALM.

### An activity map of the cerebellar cortex

What is the functional consequence of the divergent connectivity between the neocortex and cerebellum? In neocortex, preparatory activity instructing directional licking is localized to the ALM^32^. But the distribution of preparatory activity in the cerebellum has not been mapped, leaving unclear how the two brain areas organize into functional networks during motor planning of directional licking.

**Fig. 2:**
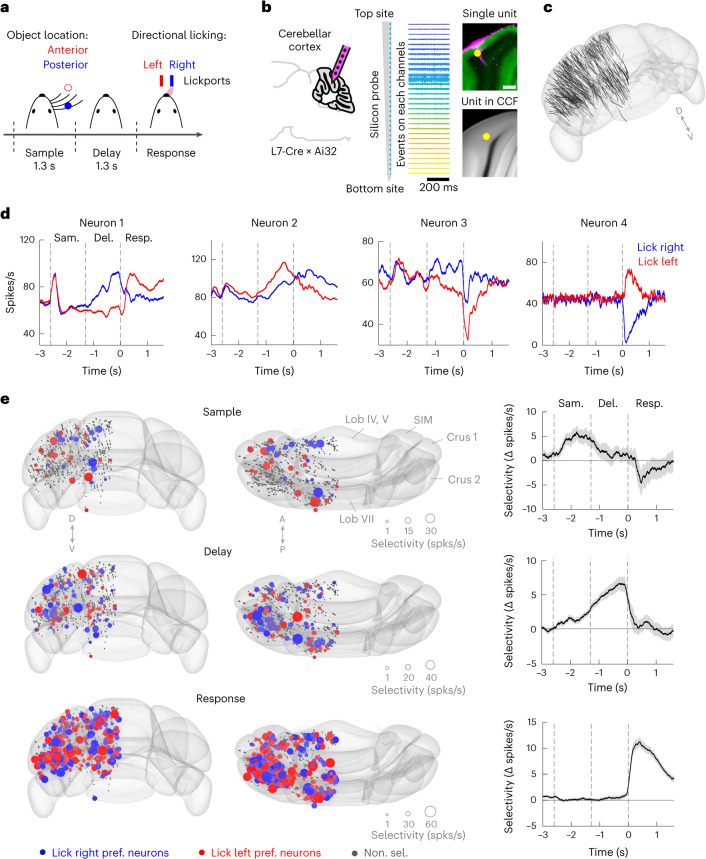
An activity map of cerebellar cortex during motor planning of directional licking. **a**, Delayed response task. The mouse reported the location of a pole by directional licking after a delay epoch. **b**, Silicon probe recording in cerebellar cortex and registration of an example unit. Left, recordings are performed in L7-cre mice crossed with Ai32 mice. The probe was painted with fluorescent dye. Middle, linear arrangement of 32 recording sites (25 µm spacing) on one probe and lamination of activity patterns in the example session. Right, labeled recording track (magenta) and the example unit (yellow dot) on raw histology image and the example unit location aligned to CCF. Green, eYFP-expressing Purkinje cells. Scale bar, 250 µm. **c**, Summary of all penetrations in CCF. *N* = 542 penetrations in 36 mice. **d**, Spike rates of four example neurons. Correct ‘lick right’ (blue) and ‘lick left’ (red) trials. Dashed lines mark behavioral epochs. **e**, Left, selectivity map of all recorded neurons during sample, delay and response epochs shown in caudal and dorsal views. Colored dots indicate neurons with significant trial-type selectivity in specific epochs. Blue indicates neurons preferring ‘lick right’; red indicates neurons preferring ‘lick left’. Dot size represents selectivity strength. Gray dots indicate nonselective neurons. Right, population selectivity (mean ± s.e.m. across neurons) of significantly selective neurons for each epoch. Selectivity is the difference in spike rate between the preferred and non-preferred trial type. Correct trials only. Trial-type preference was determined in a specific epoch using separate trials from the trials used to calculate selectivity (Methods). Averaging window, 200 ms.

**Extended Data Fig. 4:**
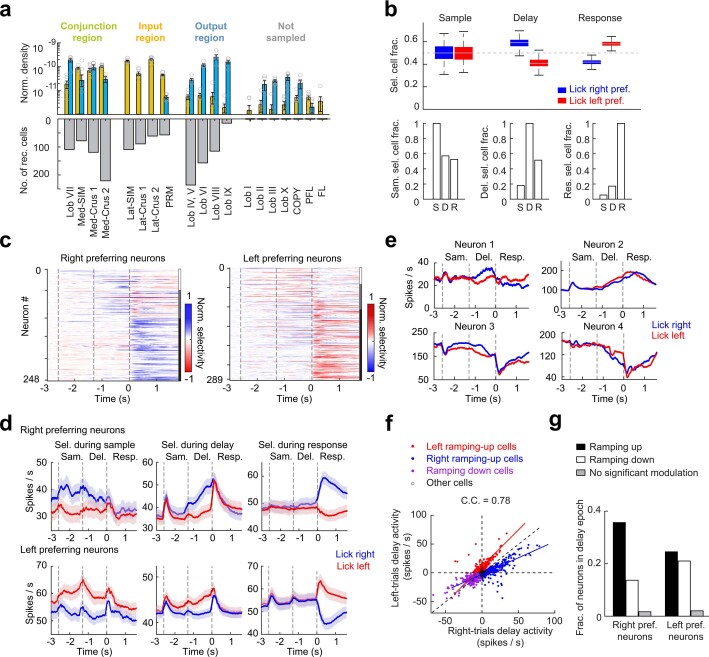
Neuronal selectivity and ramping activity in cerebellar cortex. **a**. Top, normalized ALM input density (yellow) and output Purkinje cell density (blue) of all lobules in cerebellar cortex, including lobules not sampled by silicon probe recordings. Same data as in Fig. 3b. Open circles, individual mouse (n = 7 mice for ALM input labeling; n = 5 mice for Purkinje cell labeling). Error bars, mean ± s.e.m. across mice. Bottom, recording yield in individual lobules (1366 neurons sampled from 36 mice). **b**. Top, proportion of neurons with preference for ‘lick right’ versus ‘lick left’ during different behavior epochs. Proportion is calculated among neurons with significant trial-type selectivity during each epoch (n = 42, sample epoch; n = 134, delay epoch; n = 402, response epoch). Box and whisker plot shows median, 25/75th percentiles and most extreme data points not considered as outliers (bootstrap, Methods). Bottom, for neurons with significant selectivity during each epoch, the plot shows the proportion that also exhibit significant selectivity during other epochs. S, sample epoch; D, delay epoch; R, response epoch. **c**. Population selectivity across cerebellar neurons. Selectivity is the difference in spike rate between the preferred and non-preferred trial type, normalized to the peak selectivity. Only neurons with significant trial-type selectivity are shown (‘lick right’ preferring neurons, n = 248; ‘lick left’ preferring neurons, n = 289). Neurons are sorted according to response types as indicated by the bar on the right: white, neurons exhibiting selectivity during sample or delay epoch, but not response epoch; grey, neurons exhibiting selectivity during sample or delay epoch, and response epoch; black, neurons exhibiting selectivity during only the response epoch. Dashed lines, behavioral epochs. **d**. Population response of cerebellar cortical neurons (mean ± s.e.m.) in correct ‘lick right’ trials (blue) and ‘lick left’ trials (red). Only neurons with significant trial-type selectivity during each epoch are shown (*P* < 0.01, two-tailed t-test). Top, ‘lick right’ preferring neurons. Bottom, ‘lick left’ preferring neurons. For each epoch, neurons are sorted by their preferred trial type using spike counts from 15 trials and the remaining data was used to compute the population response. **e**. Spike rates of four example neurons showing ramping activity. Neuron 1 and 2, neurons with ramping up activity. Neuron 3 and 4, neurons with ramping down activity. **f**. Distribution of delay activity in ‘lick right’ and ‘lick left’ trials for all neurons. Delay activity is the spike rate difference between the pre-sample baseline epoch and the delay epoch, calculated separately for ‘lick right’ and ‘lick left’ trials (Methods). Each dot corresponds to one neuron. Left ramping-up cells, neurons with significant ramping up activity that prefer ‘lick left’; right ramping-up cells, neurons with significant ramping up activity that prefer ‘lick right’; ramping down cells, neurons with significant ramping down activity that prefer either trial type. Other cells are shown as open circles. Line represents linear regressions (blue, right ramping-up cells; red, left ramping-up cells). C.C., correlation coefficient across all cells. **g**. Fractions of neurons with different ramping activities among ‘lick right’ and ‘lick left’ preferring neurons. All recordings are from the left hemisphere. Source data

We surveyed activity across the cerebellar cortex using silicon probe recordings during a delayed response task. Mice discriminated object location during a sample epoch and reported choice using directional licking (‘lick left’ or ‘lick right’). A delay epoch separated sensory stimulus and motor response, and mice had to use short-term memory to produce the correct licking response (Fig. 2a). We labeled recording locations with fluorescent dyes, aligned into the CCFv3 (Fig. 2b and Methods). Across 542 penetrations in 36 mice, we assembled an activity map of the cerebellar cortex (Fig. 2c and Extended Data Fig. 4a; 1,366 neurons, including identified Purkinje cells and neurons whose cell type could not be reliably inferred; Methods). Individual neurons showed diverse task-related activities, including trial-type selective activity during the delay epoch (Fig. 2d; neurons 1–3) or motor response-related activity (Fig. 2d; neuron 4). Because preparatory activity instructs lick direction^22,31,41^, we focused our analysis on neuronal selectivity differentiating ‘lick left’ and ‘lick right’ trials. Neurons with significant selectivity were sparse during the sample epoch (42/1,366), increased in number during the delay epoch (134/1,366) and became widespread across the cerebellar cortex during the motor response (402/1,366, Fig. 2e). Neurons preferring ‘lick right’ or ‘lick left’ were spatially intermingled (Fig. 2e and Extended Data Fig. 4b).

For each task epoch, we computed selectivity between individual neuron’s preferred and non-preferred trial types defined for that epoch. Sample epoch selectivity emerged during the sensory stimulus but dissipated during the delay epoch (Fig. 2e). During the delay epoch, additional neurons emerged to signal trial type (Extended Data Fig. 4b). Selectivity ramped up throughout the delay, reaching a maximum just before the motor response (Fig. 2e). Neurons with selectivity during the delay epoch (‘preparatory activity’) were distributed across the cerebellar cortex but appeared clustered in certain hotspots (Fig. 2e), including parts of Crus 1/2, simplex lobule and posterior vermal lobule VII. Preparatory activity collapsed during the response epoch (Fig. 2e): many neurons inverted their trial-type preference (for example, neurons 1 and 2; Fig. 2d), while other neurons became selective only during the motor response (for example, neuron 4; Fig. 2d and Extended Data Fig. 4c,d). In contrast to preparatory activity, selectivity during the motor response was widespread across the cerebellar cortex (Fig. 2e).

**Extended Data Fig. 5:**
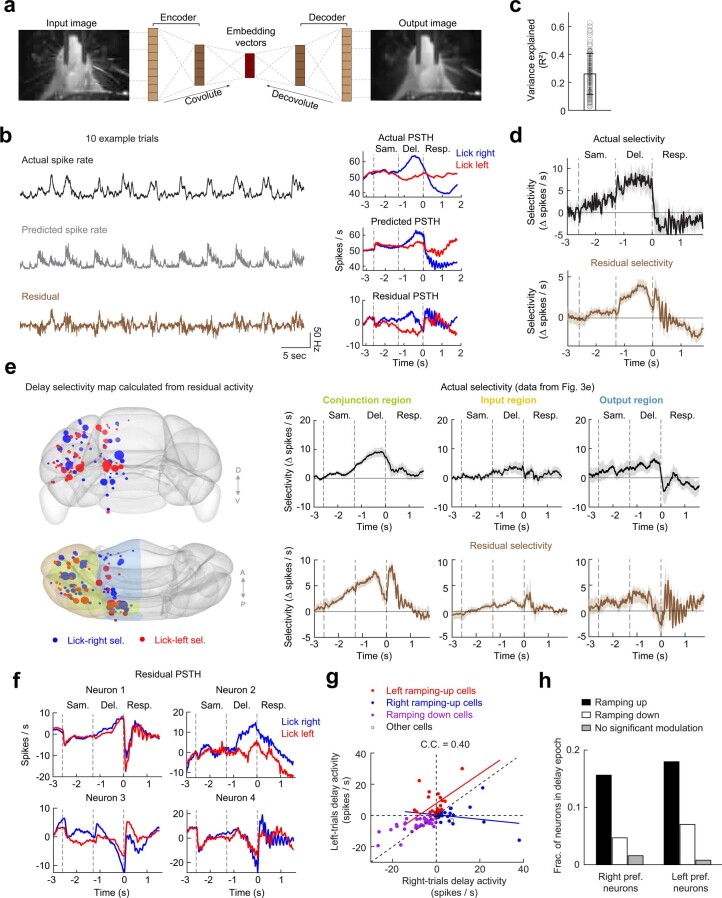
Delay epoch selectivity cannot be explained by ongoing movements. **a**. Video analysis using convolutional autoencoder. Videos of task-performing mice were compressed into a 32-dimensional embedding space, then used to predict neuronal activity through a generalized liner model (Methods). **b**. Video analysis of an example neurons. Left, single-trial spike rate (10 trials concatenated in time), predicted spike rate from video, and the residual after subtracting predicted spike rate from actual spike rate. Right, trial-aligned activity calculated from actual spike rate, predicted spike rate, and the residual. **c**. Variance explained (R^2^) by activity prediction from the video (mean ± s.d., Methods). Dots, individual neurons (n = 128). **d**. Population selectivity (mean ± s.e.m. across neurons) of neurons with significantly delay epoch selectivity. Selectivity is the difference in spike rate between the preferred and non-preferred trial type calculated using actual spike rate or residual activity after subtracting off movement predicted activity. Correct trials only. Same as Fig. 2e, but for a subset of neurons with accompanying video recordings (n = 128). **e**. Left, delay selectivity map after subtracting off movement predicted activity. Only neurons with significant selectivity during the delay epoch and with video data are included in this analysis, representing a subset of the neurons in Fig. 3a (n = 128). Blue, neurons preferring ‘lick right’; red, neurons preferring ‘lick left’. Dot size represents selectivity strength. Selectivity is calculated as the difference in spike rate between the preferred and non-preferred trial type during the delay epoch using residual activity. Right, population selectivity (mean ± s.e.m. across neurons) in conjunction regions (n = 70), input-dominant regions (n = 19), and output-dominant regions (n = 39). **f**. Four example neurons showing ramping activity in their residual activity. Neuron 1 and 2, neurons with ramping up activity. Neuron 3 and 4, neurons with ramping down activity. **g**. Same as Extended Data Fig. 4f but calculated using residual activities. **h**. Same as Extended Data Fig. 4g but calculated using residual activities.

Most neurons showing selectivity during the delay epoch also exhibited ramping activity, which gradually increased or decreased in a trial-type-specific manner (for example, ramp up, neurons 1–2; ramp down, neuron 3; Figure 2d and Extended Data Fig. 4e). The majority of neurons exhibited ramp-up activity (Extended Data Fig. 4f,g). We considered the possibility that trial-type selective activity during the delay epoch might be attributable to ongoing movements of the mice, which could differ in ‘lick left’ and ‘lick right’ trials. To address this, we built generalized linear models (GLMs) to predict neurons’ firing rate from videos of orofacial movements (Methods). Video analysis shows that ongoing movements could not explain the trial-type selectivity nor the ramping activity during the delay epoch (Extended Data Fig. 5), which suggests the activity was related to motor planning of directional licking.

These results reveal distributed preparatory and motor response-related activity across the cerebellar cortex.

### Preparatory activity is enriched in regions with conjunction of ALM input–output connectivity

**Fig. 3:**
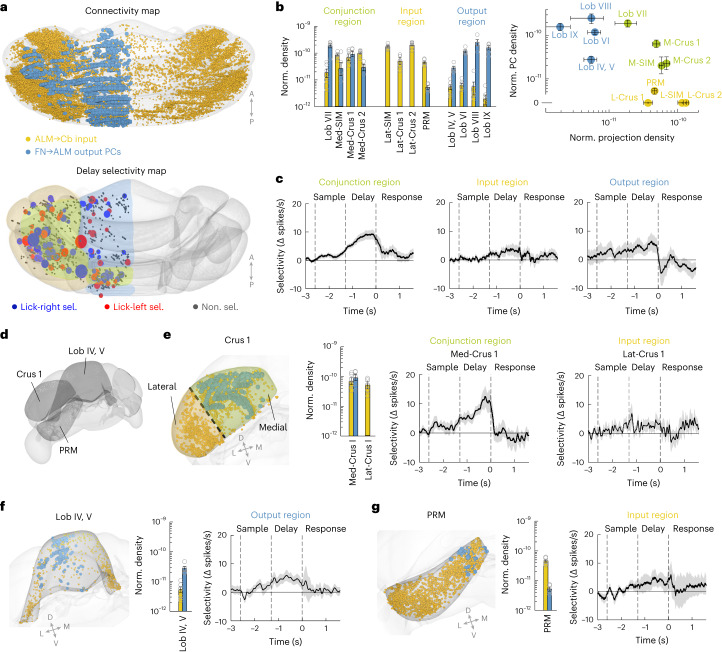
Preparatory activity is enriched in regions with conjunction of ALM input–output connectivity. **a**, Top, dorsal view of ALM input–output connectivity map. Data from Fig. 1. Yellow indicates ALM inputs; blue indicates Purkinje cells targeting the ALM via the FN. Bottom, map of delay epoch selectivity. Data from Fig. 2e. Color shading on the rendered brain indicates regions defined by ALM input–output connectivity: green indicates conjunction regions; yellow indicates input-dominant regions; blue indicates output-dominant regions (Methods). **b**, Left, normalized ALM input density (yellow) and output Purkinje cell density (blue) of individual lobules. Only lobules sampled by silicon probe recordings are shown. See Extended Data Fig. 4a for all lobules and recording yield. Simplex, Crus 1 and Crus 2 were subdivided into medial (med-SIM, med-Crus 1, med-Crus 2) and lateral (lat-SIM, lat-Crus 1, lat-Crus 2) sub-lobules based on ALM input–output connectivity. Circles indicate individual mice. Error bars denote the s.e.m. across mice. Right, ALM input density versus output Purkinje cell density for individual lobules (dots). Error bars denote the mean ± s.e.m. across mice (*n* = 7, ALM input labeling; *n* = 5 Purkinje cell labeling). **c**, Population selectivity (mean ± s.e.m. across neurons) in conjunction regions (*n* = 73), input-dominant regions (*n* = 20) and output-dominant regions (*n* = 41). Neurons with significant selectivity during the delay epoch. Selectivity is the spike rate difference between the preferred and non-preferred trial type during the delay epoch. Correct trials only. **d**, Schematics of individual lobules, Crus 1, PRM and Lob IV/V. **e**, Population selectivity within Crus 1, which has distinct patterns of ALM input–output connectivity in its subdivisions. Left, input–output connectivity pattern. Dashed line indicates the subdivision of Crus 1 into a medial portion (conjunction region) and a lateral portion (input-dominant region). Color shading is the same as in **a**. Bar plot, same as **b** but for Crus 1. Right, population selectivity (mean ± s.e.m. across neurons) of medal and lateral Crus 1. Only neurons with significant selectivity during the delay epoch (med-Crus 1, *n* = 12; lat-Crus 1, *n* = 9). **f**, Same as **e** but for population selectivity (mean ± s.e.m. across neurons) in Lob IV/V (output-dominant region). *n* = 15. **g**, Same as **e** but for population selectivity (mean ± s.e.m. across neurons) in PRM (input-dominant region). *n* = 6. Source data

To understand how the ALM and the cerebellum organize into functional circuits for motor planning of directional licking, we related the spatial distribution of preparatory activity in the cerebellar cortex to its input–output connectivity to the ALM (Fig. 3a). Preparatory activity could be enriched in regions receiving ALM inputs, which would suggest that the cerebellum inherits preparatory activity from the ALM. Alternatively, preparatory activity could be localized to regions providing outputs to the ALM, which would suggest preparatory activity is passed from the cerebellum to the ALM.

We organized the cerebellar cortex into regions that: (1) received ALM inputs but provided little outputs to the ALM (‘input-dominant regions’); (2) received little ALM inputs but provided outputs to ALM (‘output-dominant regions’); and (3) exhibited a conjunction of input–output connectivity to ALM (‘conjunction regions’, Fig. 3a,b and Methods). We first divided the cerebellar cortex into individual lobules. Lobules Crus 1/2 and simplex showed distinct patterns of ALM connectivity within lobule, where the medial portions exhibited a conjunction of input–output connectivity and the lateral portions mainly exhibited input connectivity. For these lobules, we subdivided them into medial and lateral sub-lobules according to their distinctive connectivity profiles (med-SIM, med-Crus 1 and med-Crus 2 versus lat-SIM, lat-Crus 1 and lat-Crus 2). Lobules and sub-lobules were then grouped based on their anatomical connectivity (Fig. 3b and Methods).

**Extended Data Fig. 6:**
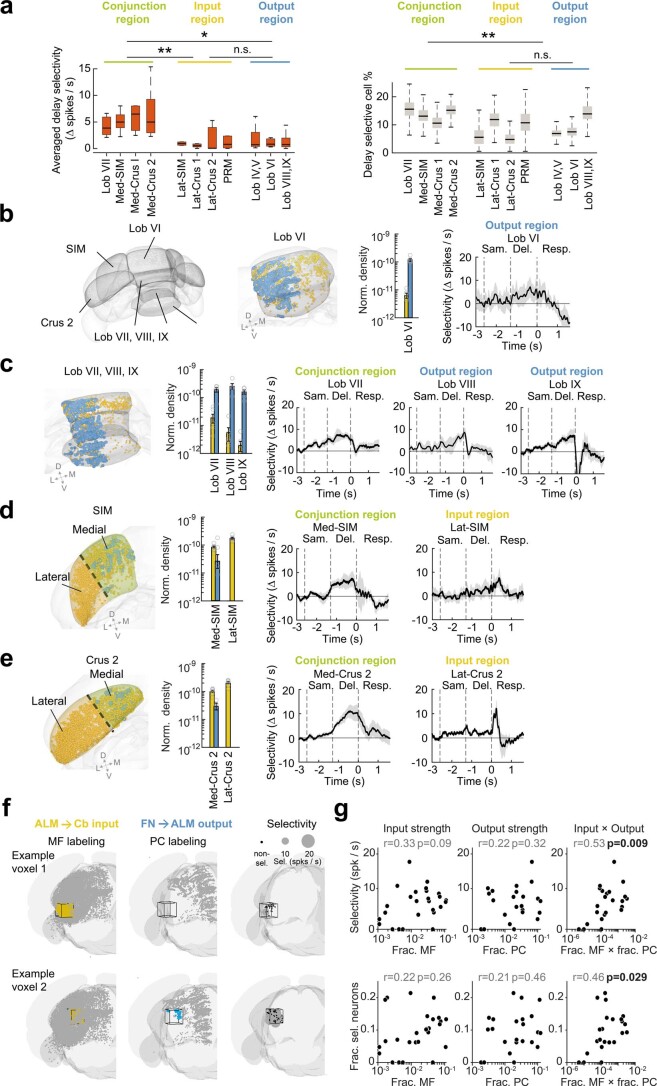
Distribution of preparatory activity coincides with reciprocal connectivity with ALM via FN. **a**. Left, selectivity of individual lobules during the delay epoch in conjunction regions, input-dominant regions, and output-dominant regions. Neurons with significant selectivity during the delay epoch. Box and whisker plot shows median, 25/75th percentiles and most extreme data points not considered as outliers. Kruskal-Wallis One-Way ANOVA Test, H(2) = 12.02, *P* = 0.0025. Post-hoc pair-wise Mann Whitney U Test between conjunction vs. input-dominant, **P = 0.0013, conjunction vs. output-dominant, *P = 0.027. For statistical comparison between conjunction, input-, and output-dominant regions, the individual lobules in each region are combined (n = 71, 22 and 40 delay selective neurons from conjunction, input and output regions). Right, proportions of neurons with significant selectivity during the delay epoch in individual lobules. Box and whisker plot shows median, 25/75th percentiles and most extreme data points not considered as outliers (bootstrap, Methods). *Χ*^2^(1) = 7.392, ** *P* = 0.007, Chi-squared test. **b**. Left, Schematics of individual lobules. Right, population selectivity within lobule VI (output-dominant region). From left to right, schematic of Lob VI in cerebellum; ALM input-output connectivity pattern within Lob VI (same as in Fig. 3b); population selectivity (mean ± s.e.m. across neurons). Only neurons with significant selectivity during the delay epoch are used for selectivity calculation (n = 11). **c**. Same as **b** for lobule VII (conjunction region, n = 15), lobule VIII (output-dominant region, n = 12), and lobule IX (output-dominant regions, n = 3). Only neurons with significant selectivity during the delay epoch are used for selectivity calculation. Selectivity was shown as mean ± s.e.m. across neurons. **d**. Same as **b** for simplex. Dashed line, simplex lobule is subdivided into a medial portion (conjunction region, n = 17) and a lateral portion (input-dominant region, n = 2). Only neurons with significant selectivity during the delay epoch are used for selectivity calculation. Selectivity was shown as mean ± s.e.m. across neurons. **e**. Same as **b** for Crus 2. Crus 2 is subdivided into a medial portion (conjunction region, n = 29) and a lateral portion (input-dominant region, n = 3). Only neurons with significant selectivity during the delay epoch are used for selectivity calculation. Selectivity was shown as mean ± s.e.m. across neurons. **f**. Tessellating the cerebellum into voxels. The plots show two example voxels. Mossy fiber terminals (yellow), Purkinje cell labeling (blue), and units recorded (black dots) within each voxel. Significant selectivity during the delay epoch is indicated by gray dot. Voxels 2 shows stronger preparatory activity than voxel 1. **g**. Preparatory activity correlates only with conjunction of input and output connectivity. Dots, individual voxels. Only voxels containing 5 or more neurons are included. Pearson’s correlation and p values (two-sided test) are shown. Source data

Interestingly, preparatory activity was specifically enriched in the conjunction regions. A higher fraction of neurons in the conjunction regions exhibited trial-type selectivity during the delay epoch compared to the input-dominant and output-dominant regions (Extended Data Fig. 6a; conjunction versus input-dominant and output-dominant regions, Chi-squared test*, Χ*^2^(1) = 7.392, *P* = 0.007). Among the selective neurons, the conjunction regions also exhibited stronger selectivity compared to the input-dominant and output-dominant regions (Fig. 3c and Extended Data Fig. 6a; Kruskal–Wallis one-way analysis of variance (ANOVA), *H*(2) = 12.02, *P* = 0.0025; post hoc Mann–Whitney *U* test, conjunction versus input-dominant regions, *P* = 0.0013; conjunction versus output-dominant regions, *P* = 0.027). This observation was further supported when examining individual lobules (Fig. 3e–g and Extended Data Fig. 6b–e). For Crus 1/2 and simplex that showed distinct patterns of ALM connectivity within lobule, the medial portions with conjunctive input–output connectivity exhibited stronger delay epoch selectivity than the lateral portions.

We additionally used a more continuous method to relate ALM input–output connectivity to preparatory activity. In CCFv3, we tessellated the cerebellar cortex into 100 × 100 × 100 µm voxels without regard to lobule boundaries (Extended Data Fig. 6f and Methods). Within each voxel, we quantified input connectivity as the number of labeled mossy fiber terminals and output connectivity as the number of labeled Purkinje cells. We quantified preparatory activity as the fraction of neurons exhibiting significant trial-type selectivity during the delay epoch, and average selectivity amplitude among the selective neurons. Both measures of preparatory activity were better predicted by the product of input and output connectivity than either connectivity alone (Extended Data Fig. 6g). These analyses show that the distribution of preparatory activity in the cerebellar cortex is correlated with reciprocal connectivity with the ALM.

**Extended Data Fig. 7:**
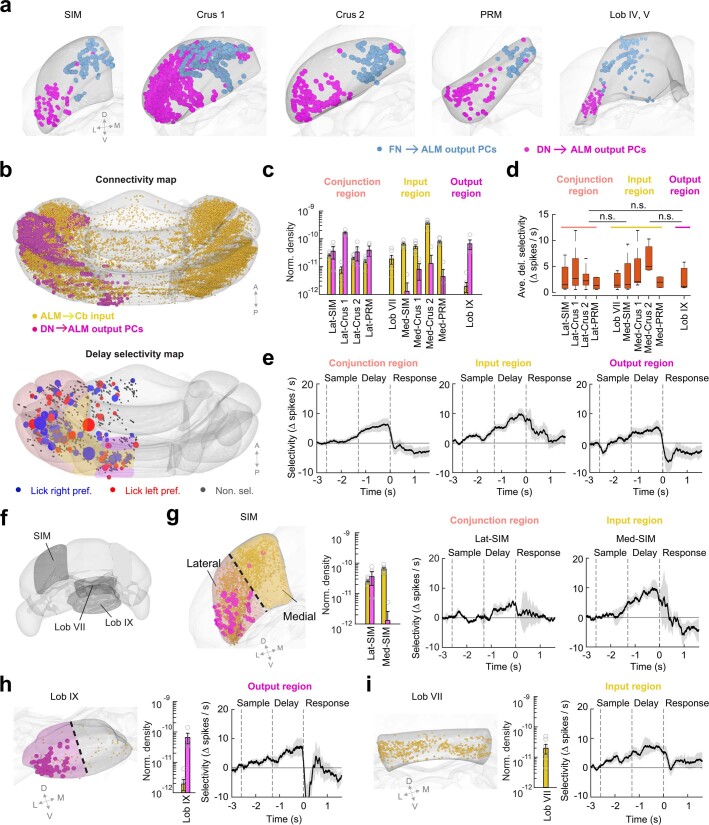
Distribution of preparatory activity does not coincide with reciprocal connectivity with ALM via DN. **a**. Distribution of Purkinje cells targeting ALM via FN (blue, data from 5 mice) and DN (magenta, data from 4 mice) in example lobules. DN-projecting Purkinje cells are located more laterally than FN-projecting Purkinje cells. In Crus 1, the two types of Purkinje cells are spatially overlapping. **b**. Top, dorsal view of ALM input-output connectivity map via DN. Data from Fig. 1e and Extended Data Fig. 3h. Yellow, ALM input map (data from 7 mice); magenta, Purkinje cells targeting ALM via DN (data from 4 mice). Bottom, activity map of delay epoch selectivity. Data from Fig. 2e. Color shading on the rendered brain indicated regions defined by ALM input-output connectivity: pink, conjunction regions; yellow, input-dominant regions; magenta, output-dominant regions (see Methods). **c**. Normalized ALM input density (yellow) and output Purkinje cell density (magenta) of individual lobules in conjunction regions, input-dominant regions, and output-dominant regions. Only lobules sampled by silicon probe recordings are shown. Lobule simplex, Crus 1, Crus 2, and paramedial lobule are subdivided into medial (med-SIM, med-Crus 1, med-Crus 2, med-PRM) and lateral (lat-SIM, lat-Crus 1, lat-Crus 2, lat-PRM) portions based on ALM input-output connectivity via the dentate nucleus. Note that sub-lobule boundaries here are defined based on connectivity via the dentate nucleus (Methods), thus the sub-lobules are different from those in Fig. 3 and Extended Data Fig. 6. Open circles, individual mice (n = 7 mice for ALM input labeling; n = 4 mice for Purkinje cell labeling via DN). Error bars, s.e.m. across mice. **d**. Selectivity of individual lobules during the delay epoch in conjunction regions, input-dominant regions, and output-dominant regions. Neurons with significant selectivity during the delay epoch. Kruskal-Wallis One-Way ANOVA Test, H(2) = 2.84, *P* = 0.2416. For statistical comparison between conjunction, input-, and output-dominant regions, the individual lobules in each region are combined (n = 63, 30 and 3 delay selective neurons from conjunction, input and output regions). Box and whisker plot shows median, 25/75th percentiles and most extreme data points not considered as outliers. **e**. Population selectivity (mean ± s.e.m. across neurons) in conjunction regions (n = 55), input-dominant regions (n = 38), and output-dominant regions (n = 3). Only neurons with significant selectivity during the delay epoch are used for selectivity calculation. Selectivity is the difference in spike rate between the preferred and non-preferred trial type during the delay epoch. Correct trials only. Distribution of selectivity does not coincide with conjunction region. **f**. Schematics of individual lobules, lobule simplex, lobule VII and lob IX. **g**. Population selectivity within lobule simplex which has distinct patterns of ALM input-output connectivity within lobule. Left, input-output connectivity pattern (same as in **b** top but in oblique view). Dashed line, lobule simplex is subdivided into a medial portion (input-dominant region) and a lateral portion (conjunction region). Color shading is the same as in **b**. Bar plot, same as **c** but only showing lobule simplex. Right, population selectivity (mean ± s.e.m. across neurons) of medial and lateral lobule simplex. Neurons with significant selectivity during the delay epoch (medial SIM, n = 8; lateral SIM, n = 11). Distribution of selectivity does not coincide with conjunction region. **h**. Same as **g** but for population selectivity (mean ± s.e.m. across neurons) in Lob IX (output-dominant region). n = 3. **i**. Same as **g** but for population selectivity (mean ± s.e.m. across neurons) in lobule VII (input-dominant region). n = 15. Source data

Is preparatory activity selectively enriched in the conjunction regions defined by cerebellar output via the fastigial nucleus? Cerebellar outputs also target the thalamus via the dentate nucleus, including parts of the ALM-projecting thalamus^11,13^. To clarify the topography of preparatory activity in relation to distinct cerebellar output pathways, we redefined the output regions based on the Purkinje cells retrogradely labeled from the thalamus-projecting dentate neurons (Extended Data Fig. 7 and Methods). The output regions defined by the dentate pathway shared a similar topography with ALM input regions (Extended Data Fig. 7b), forming a parallel cortico-cerebellar loop with the ALM besides the fastigial pathway. Yet, when the output regions were defined by the dentate pathway, the delay epoch selectivity was no longer localized to the conjunction regions (Extended Data Fig. 7d; Kruskal–Wallis one-way ANOVA, *H*(2) = 1.61, *P* = 0.447). Thus, preparatory activity in the cerebellar cortex appears to specifically coincide with its reciprocal connectivity to ALM via the fastigial nucleus.

Together, these data revealed a close correspondence between conjunctive ALM input–output connectivity and distribution of preparatory activity in the cerebellum. This suggests that the cerebellum is not simply downstream or upstream of the ALM. Rather, the maintenance of preparatory activity depends on reciprocal communication between the ALM and cerebellar cortex.

### Conjunction regions are required for motor planning of directional licking

**Fig. 4:**
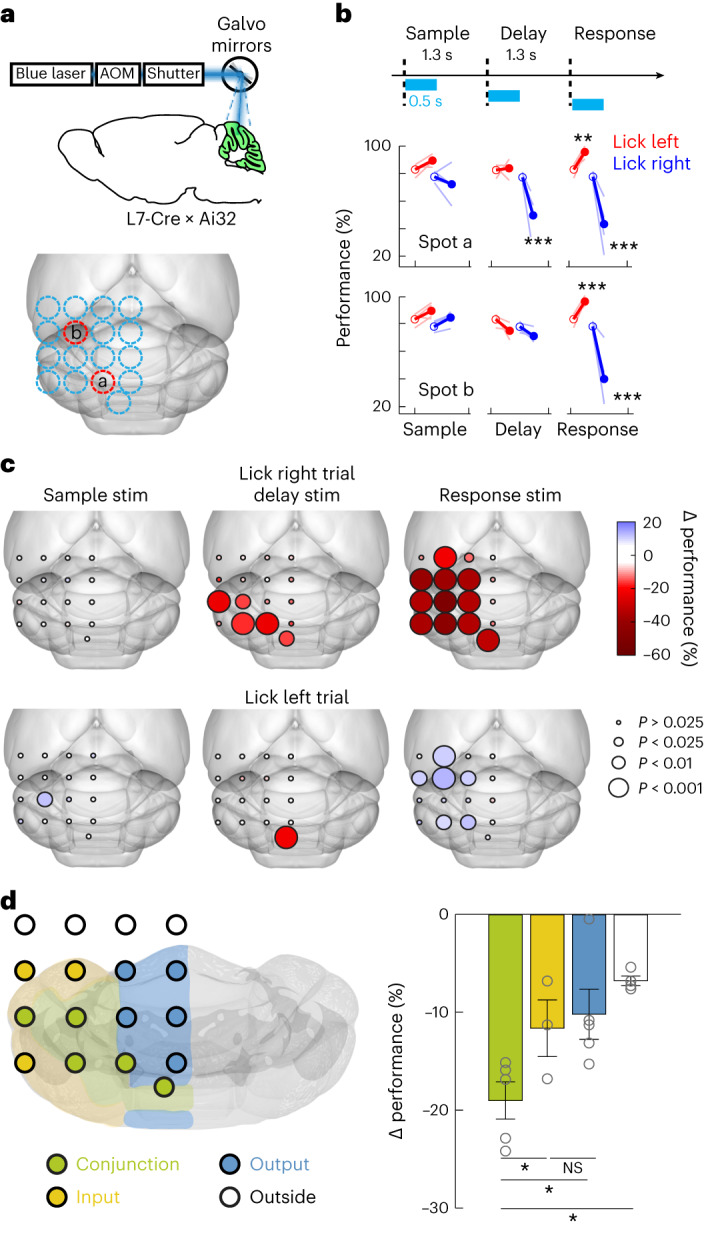
Regions with conjunction of ALM input–output connectivity contribute to motor planning behavior. **a**, Top, photostimulation is delivered using a scanner laser through a clear-skull implant. Bottom, a grid of 4 × 4 photostimulation locations (1-mm spacing). See alignment of the photostimulation locations into the CCF in Extended Data Fig. 8a. The photostimulation is over the left hemisphere. Each location was chosen randomly for photostimulation during the sample, delay or response epoch (*n* = 4 mice). In separate experiments, we tested one posterior location outside the 4 × 4 grid (posterior 4.1 mm, lateral 0.5 mm from lambda, *n* = 7 mice). Red circles indicate the two example photostimulation locations shown in **b**. **b**, Top, task and photostimulation timeline. Bottom, performance (percentage correct) after photostimulation in two example spots during specific epochs. ‘Lick left’ (red) and ‘lick right’ (blue) trials were grouped by instructed lick directions. Thick lines indicate the mean; thin lines indicate individual mice (*n* = 4). Open circles indicate performance in control trials; solid dots indicate photostimulation trials. Lick right trials, spot a, ***P = 0.00059 in delay epoch, ***P = 0.00043 in response epoch; spot b, ***P = 0; lick left trials, spot a, **P = 0.0185 in response epoch. Bootstrap, one-sided test, adjusted for multiple comparisons (Methods). **c**, Cerebellar regions involved in delayed response task during sample, delay and response epochs. Color codes for the change in performance (percentage correct) under photostimulation relative to control (∆ performance). Performances in ‘lick right’ (top row) and ‘lick left’ (bottom row) trials are shown separately. Trials across all photostimulation powers were combined (1–4 mW; Methods). Dot size codes for significance from bootstrap (Methods). For the 4 × 4 grid photostimulation, *n* = 4 mice; for the posterior spot photostimulation, *n* = 7 mice. **d**, Effect of delay epoch photostimulation across cerebellar regions defined by ALM input–output connectivity. Left, the photostimulation spots were grouped into conjunction regions (green dots), input-dominant regions (yellow dots) or output-dominant regions (blue dots). White dots indicate spots outside the cerebellum. Right, averaged ∆ performance in ‘lick right’ trials. Kruskal–Wallis one-way ANOVA, *H*(3) = 13.22, *P* = 0.0041. Post hoc pair-wise Mann–Whitney *U* test, conjunction versus input-dominant, *P = 0.034; conjunction versus output-dominant, *P = 0.016; conjunction versus outside regions, *P = 0.016. Error bars indicate the s.e.m. across photostimulation spots. Conjunction region, 5 spots from 11 mice; input region, 3 spots from 4 mice; output region, 5 spots from 4 mice; outside region, 4 spots from 4 mice. The slight performance decrease when photostimulation occurred outside the cerebellum may be caused by light scattering through the intact skull, which may affect parts of the cerebellum. NS, not significant. Source data

**Extended Data Fig. 8:**
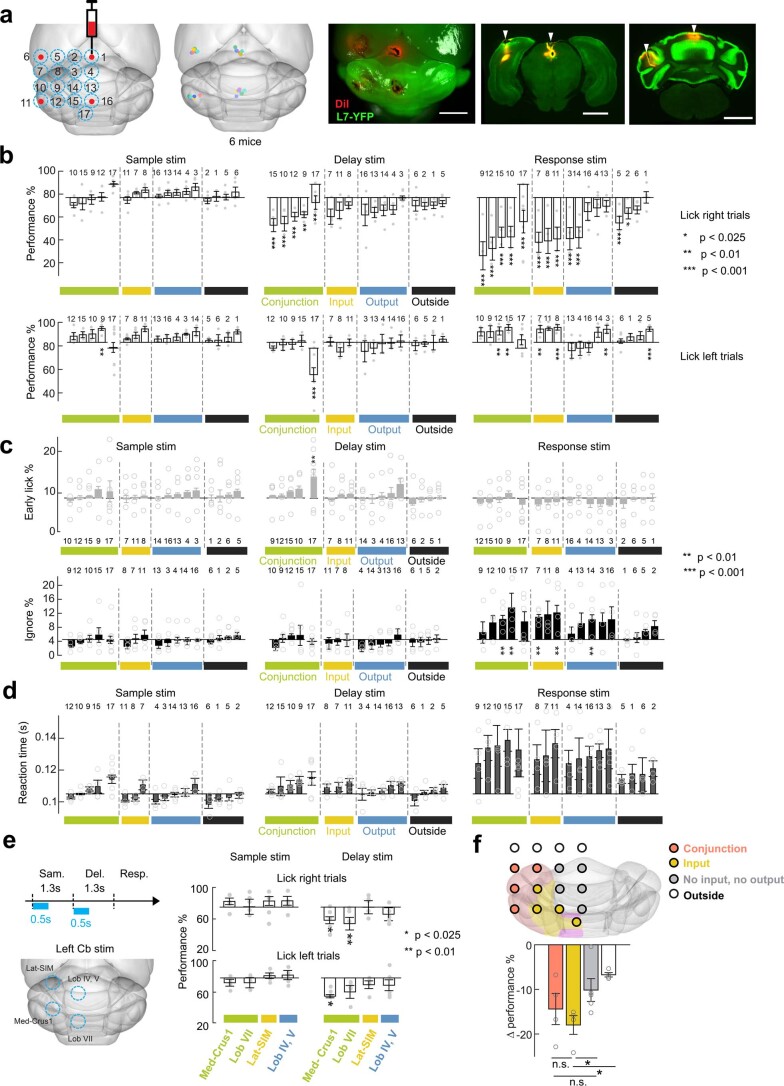
Involvement of cerebellar regions in delayed response task. **a**. Alignment of photostimulation locations into CCF. Left, DiI is injected in four corners of the photostimulation grid to localize the grid in CCF (Methods). Coronal sections with DiI labeling are aligned into CCF to anchor the grid. The remaining spots are placed into CCF by interpolation. Labeled grid corners on the template brain are shown for 6 mice. Color codes for different mice. Right, image of an example brain showing DiI injection sites from top view and two example coronal sections after alignment into CCF. Scale bars in all images, 2 mm. **b**. Performance (% correct) when photostimulating different locations during sample, delay, or response epochs. The number above each bar indicates the stimulation spot in **a**. Spot #17 is outside of the 4x4 grid and is tested in separate experiments. Photostimulation locations are grouped by ALM input-output connectivity via the fastigial nucleus (indicated by colored bar on the bottom, same color scheme as Fig. 4d). The slight performance decrease when photostimulating outside of the cerebellum may be caused by light scattering through the intact skull, which may affect parts of the cerebellum. Performances are plotted as bars from an offset that indicate mean performance in control trials. Performance in ‘lick right’ and ‘lick left’ trials are computed separately. Error bars, mean ± s.e.m. from bootstrap (Methods). Lick right trials delay epoch, spot #15 ***P = 0.00059, #10 ***P = 0.00027, #12 ***P = 0.00025, #9, **P = 0.00265, #17 **P = 0.00621; response epoch, spot #9 ***P = 0, #12 ***P = 0, #15 ***P = 0.00043, #10 ***P = 3 × 10^−5^, #17 ***P = 1 × 10^−5^, #7 ***P = 0, #8 ***P = 0, #11 ***P = 0.00093, #3 ***P = 0, #14 ***P = 0, #5 ***P = 0.00021, #2 *P = 0.01304. Lick left trials sample epoch, spot #9 **P = 0.00125; delay epoch, spot #17 ***P = 2 × 10^−5^; response epoch, spot #12 **P = 0.0087, #15 **P = 0.00185, #7 **P = 0.00387, #8 ***P = 0.00035, #3 **P = 0.00464, #5 ***P = 0.00067.Dots indicate individual mouse (n = 4 mice for 4x4 grid photostimulation; n = 7 mice for spot #17 photostimulation). **c**. Same as **b** for early lick rate and ignore rate. ‘Lick right’ and ‘lick left’ trials are combined. Offsets are the mean early lick rate and ignore rate in control trials. Early lick rate, spot 17 **P = 0.006, Ignore rate, spot #10 **P = 0.002, #15 **P = 0.004, #7 **P = 0.001, #8 **P = 0.0012, #14 **P = 0.0011. Error bars, mean ± s.e.m. from bootstrap (Methods). Open circles indicate individual mouse (n = 4 mice for 4x4 grid photostimulation; n = 7 mice for spot #17 photostimulation). **d**. Same as **b** for reaction time change. Reaction time is the time between response cue and first lick. ‘Lick right’ and ‘lick left’ trials are combined. Offsets show the mean reaction time in control trials. Error bars, mean ± s.e.m. from bootstrap (Methods). Open circles indicate individual mouse (n = 4 mice for 4x4 grid photostimulation; n = 7 mice for spot #17 photostimulation). **e**. Left, optogenetic experiment perturbing med-Crus 1 (a conjunction regions), Lob VII (a conjunction regions), lat-SIM (an input-dominant region) and Lob IV, V (an output-dominant region) in the same session to allow a side-by-side comparison. In a subset of trials, photostimulation is delivered randomly to one of the four spots during either sample or delay epoch. Right, performance (% correct) when photostimulating different locations in sample and delay epochs(n = 5 mice). Lick right trials *P = 0.017, **P = 0.006; lick left trials *P = 0.012p. Error bars, mean ± s.e.m. from bootstrap (Methods). **f**. Effect of delay epoch photostimulation across cerebellar regions does not coincide with ALM input-output connectivity via the dentate nucleus. Top, conjunction regions (pink spots), input-dominant regions (yellow spots), regions with no input or output connectivity (grey spots), spots outside of the cerebellum (white spots). No photostimulation spots covered the output-dominant region (magenta region on the rendered brain). Bottom, averaged ∆performance in ‘lick right’ trials. Kruskal-Wallis One-Way ANOVA Test, H(3) = 10.81, *P* = 0.0128. * Post-hoc Mann Whitney U Test between input-dominant vs. no connectivity regions, *P = 0.032; input-dominant vs. outside regions, *P = 0.029. Error bars, s.e.m. across photostimulation spots within each region. Conjunction region, 3 spots from 4 mice; input region, 4 spots from 11 mice; no connectivity region, 5 spots from 4 mice; outside region, 4 spots from 4 mice. Source data

Preparatory activity is enriched in cerebellar regions with conjunctive input–output connectivity to the ALM, but do these regions contribute to motor planning of directional licking? We surveyed the dorsal cerebellar cortex for involvement in the motor planning behavior by disrupting activity using channelrhodopsin-2 (ChR2) activation of Purkinje cells (L7-cre mice crossed with Ai32 mice; Methods). We tested 16 evenly spaced spots across the dorsal cerebellar cortex using a scanning laser (Fig. 4a; 4 mice, 153 sessions, 57,502 trials, 1,520 ± 161 photostimulation trials per spot, mean ± s.d.)^31^, covering most of the input-dominant, output-dominant and conjunction regions (Extended Data Fig. 8a). In a separate experiment, we additionally tested a spot over the posterior vermis (a conjunction region) because the posterior cerebellar cortex was inaccessible from the dorsal surface (6 mice, 31 sessions, 11,398 trials, 4,782 photostimulation trials). In each trial, we transiently perturbed activity in a single spot during the beginning of the sample, delay or response epoch (Fig. 4b).

Photostimulation biased upcoming choice in an epoch-dependent and region-dependent manner. During the sample epoch, photostimulation of most cerebellar regions did not induce significant changes in task performance (Fig. 4b,c). This mirrored the lack of trial-type selective activity in the cerebellum during the sample epoch (Fig. 2e). During the delay epoch, perturbation in some spots biased upcoming lick direction, which led to incorrect choices (Fig. 4b, spot a), while perturbing other spots caused minimal effect (Fig. 4b, spot b). A significant performance decrease was induced in parts of Crus 1/2, simplex lobule and posterior vermis (Fig. 4c), which coincided with the regions enriched in preparatory activity (Fig. 2e). For most spots, photoactivation of the left cerebellar cortex biased future lick direction to the left, resulting in decreased performance in the ‘lick right’ trials (Fig. 4c and Extended Data Fig. 8b). Photoactivation of the posterior vermis biased lick direction to either left or right in individual mice, resulting in decreased performance in both trial types (Fig. 4c and Extended Data Fig. 8b). Photostimulation during the early delay epoch biased future lick direction even though the photostimuli ceased 800 ms before the motor response, consistent with a disruption of motor planning.

During the response epoch, photoactivation of most cerebellar regions biased lick direction to the left, resulting in decreased performance in the ‘lick right’ trials and increased performance in the ‘lick left’ trials (Fig. 4c and Extended Data Fig. 8b). Photoactivation also increased the reaction time of the first lick (Extended Data Fig. 8d). In some trials, photoactivation blocked licking response altogether, resulting in increased ignore rate (Extended Data Fig. 8c). These results suggest an additional role of the cerebellum in licking motor control^28,33,34^. Notably, whereas specific regions of the cerebellar cortex were necessary for task performance during the delay epoch, more distributed regions of the cerebellar cortex were required for the licking motor response (Fig. 4c and Extended Data Fig. 8b–d).

To examine the topography of cerebellar regions contributing to motor planning of directional licking, we aligned the photostimulation spots into CCFv3 (Extended Data Fig. 8a) and compared the effect of delay epoch photostimulation across the input-dominant, output-dominant and conjunction regions (Fig. 4d). Photostimulation of the conjunction regions induced the strongest effect on upcoming lick direction (Fig. 4d; Kruskal–Wallis one-way ANOVA, *H*(3) = 13.22, *P* = 0.0041; post hoc pair-wise Mann–Whitney *U* test between conjunction versus input-dominant, *P* = 0.034; conjunction versus output-dominant, *P* = 0.016; conjunction versus outside regions, *P* = 0.016). This suggests that cerebellar involvement in motor planning of directional licking is related to its reciprocal connectivity to the ALM. To further confirm the involvement of conjunction regions, we performed an additional experiment in which we tested two conjunction regions (posterior vermis and medial Crus 1/2), an input-dominant region (lateral simplex) and an output-dominant region (anterior vermis) in the same session to allow a side-by-side comparison (Extended Data Fig. 8e; 5 mice, 42 sessions). Delay epoch perturbation of either conjunction region induced stronger reduction in task performance than the input-dominant or output-dominant region (Extended Data Fig. 8e). Finally, the cerebellar regions affecting task performance were misaligned with its ALM input–output connectivity via the dentate nucleus (Extended Data Fig. 8f). Thus, cerebellar involvement in motor planning of directional licking appears to specifically coincide with its reciprocal connectivity to the ALM via the fastigial nucleus.

These data show that cerebellar regions with reciprocal connectivity to the ALM are required for motor planning of directional licking.

### Conjunction regions maintain preparatory activity

**Fig. 5:**
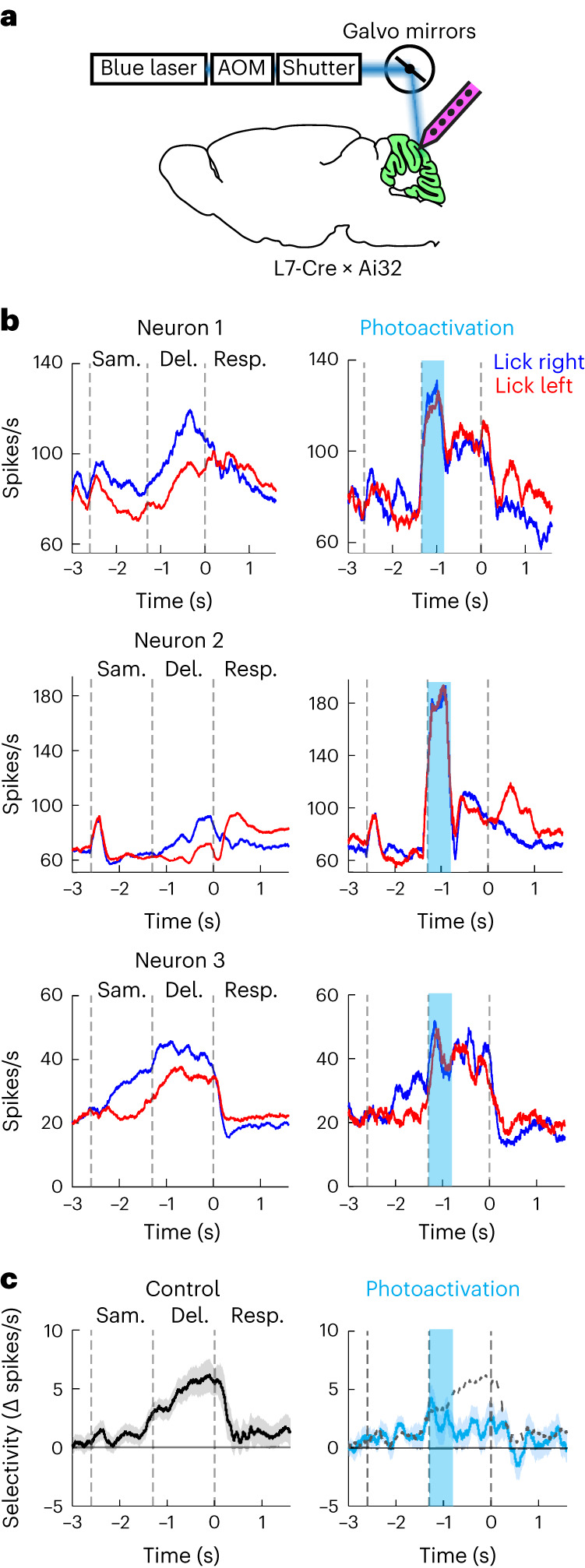
Purkinje cells in conjunction regions are required for maintaining preparatory activity. **a**, Electrophysiology recording and optogenetic perturbations in L7-cre mice crossed with Ai32 during behavior. **b**, Spike rates of three example neurons in control trials (left) and in photostimulation trials (right). Blue indicates ‘lick right’ trials; red indicates ‘lick left’ trials. Dashed lines mark behavioral epochs. **c**, Population selectivity (mean ± s.e.m. across neurons) in conjunction regions. Selectivity is the difference in spike rate between the preferred and non-preferred trial types during the delay epoch. Left, control; right, photostimulation trials. All neurons with significant selectivity during the delay epoch and that were tested for >3 trials in all conditions were included (*n* = 88). The dashed line represents the mean from control trials.

If preparatory activity is orchestrated by reciprocal communication between the ALM and cerebellar conjunction regions, transiently breaking this communication should disrupt preparatory activity. We tested this hypothesis by recording preparatory activity while transiently perturbing the Purkinje cells in the conjunction regions (L7-cre mice crossed with Ai32 mice; Methods). We first recorded from the perturbed conjunction regions to examine the direct effect of photostimulation (Fig. 5a). In control trials, individual neurons exhibited trial-type selectivity during the delay epoch, which was abolished after a transient perturbation during the early delay epoch (Fig. 5b). Across the population, delay epoch selectivity was persistently abolished by transiently perturbing the conjunction regions (Fig. 5c). This suggests that Purkinje cells in the conjunction regions are required for the maintenance of preparatory activity.

**Fig. 6:**
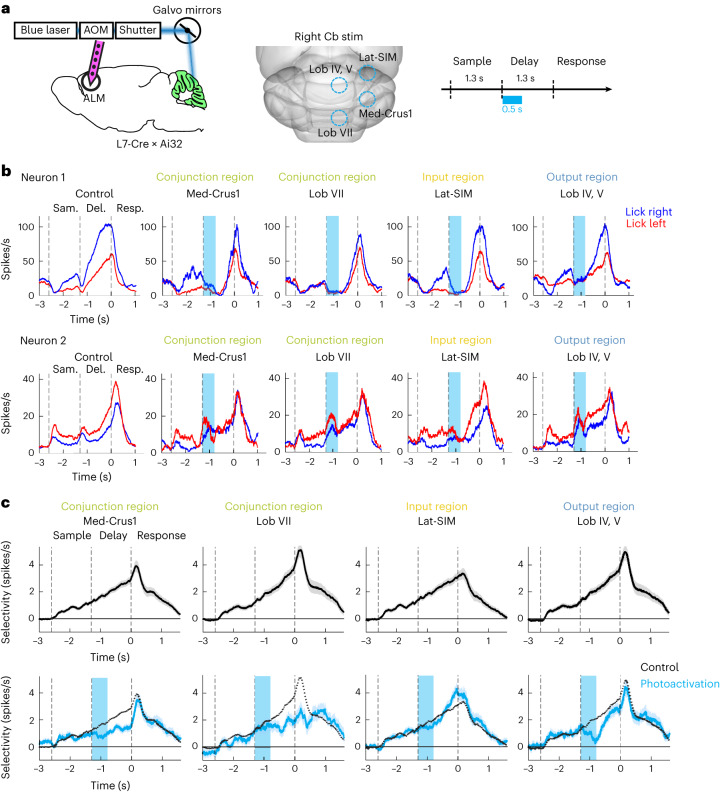
Cerebellar conjunction regions selectively affect ALM preparatory activity. **a**, Left, electrophysiology recording in the ALM (bilateral) during optogenetic perturbations of distinct cerebellar regions in L7-cre mice crossed with Ai32 mice. Middle, photostimulation locations (blue circles). Photostimulation is always on the right hemisphere. Right, task and photostimulation timeline. **b**, Spike rates of two example ALM neurons under control (left) and different photostimulation conditions (right). Blue, ‘lick right’ trials; red, ‘lick left’ trials. Dashed lines mark behavioral epochs. **c**, ALM population selectivity under control (top) and photostimulation conditions (bottom). Mean ± s.e.m. across neurons. The dashed line represents the mean from control trials. Selectivity is the difference in spike rate between the preferred and non-preferred trial type during the delay epoch. For each photostimulation condition, all neurons with significant selectivity during the delay epoch and that were tested for >3 trials in control and photostimulation conditions were included (*n* = 213, 250, 314 and 203 for lobule IV/V, lateral simplex, medial Crus 1/2 and lobule VII, respectively). Photostimulation produced similar effects across both ALM hemispheres; thus, data from both ALM hemispheres were pooled.

We next examined the impact of cerebellar conjunction regions on ALM preparatory activity (Fig. 6a), specifically medial Crus 1/2 and lobule VII (Methods). For comparison, we also perturbed an input-dominant region, the lateral simplex and an output-dominant region, lobules IV and V. Perturbing all cerebellar regions produced activity changes in the ALM (Fig. 6b), even in input-dominant regions that do not provide direct output back to the ALM (Fig. 6b; lateral simplex). These light-induced activity changes suggest that broad regions of the cerebellum could influence ALM activity through either direct or indirect pathways. The ALM receives inputs from other neocortical regions^26,29^, which may be influenced by cerebellar regions outside the output regions mapped here. However, perturbing different cerebellar regions had distinct effects on ALM preparatory activity. A transient perturbation in the conjunction regions during the early delay epoch persistently reduced trial-type selectivity in ALM (Fig. 6c). In contrast, selectivity rapidly recovered after perturbations in the input-dominant or output-dominant regions (Fig. 6c). Therefore, although the ALM received converging inputs from large territories of the cerebellum, the conjunction regions specifically influenced trial-type selective activity.

Together, these data show that cerebellar conjunction regions selectively form a functional closed loop with the ALM to maintain preparatory activity.

### Preparatory activity is maintained by Purkinje cell simple spikes but not complex spikes

Purkinje cells integrate two distinct sources of inputs to produce output patterns to the cerebellar nuclei. Inputs from the pons (via mossy fibers and granule cells) drive high-frequency simple spikes (SS). Inputs from the inferior olive (via climbing fibers) trigger complex spikes (CS) that can regulate SS outputs^42–45^. Activity preceding instructed movements has been reported in both the mossy fiber pathway^6,12,24^ and the climbing fiber pathway^46–48^. However, the relative contributions of the two pathways to preparatory activity remain unknown.

**Fig. 7:**
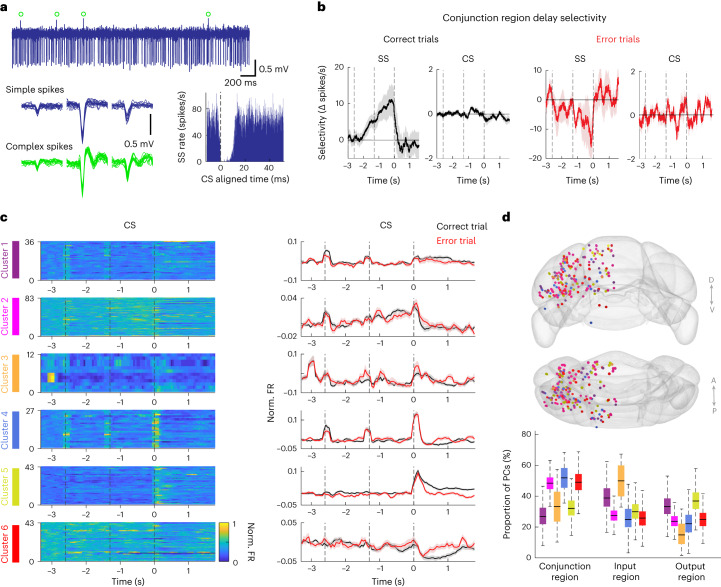
Preparatory activity is maintained by Purkinje cell SS but not CS. **a**, Recording from an example Purkinje cell. Top, raw voltage trace. Green circles mark the CS. Bottom left, waveforms of SS (blue) and CS (green) from three consecutive channels. Bottom right, cross-correlogram between SS and CS. The characteristic pause in SS after a CS confirms that these spikes originate from the same Purkinje cell. **b**, Population selectivity (mean ± s.e.m.) of Purkinje cells in conjunction regions. Selectivity of SS or CS in correct trials (black) and error trials (red). Selectivity is the spike rate difference between the preferred and non-preferred trial types. Trial-type preference was determined by SS activity during the delay epoch in correct trials. Separate trials were used to determine trial-type preference from the trials used to calculate selectivity (Methods). Trial type was defined by instructed lick directions. In error trials, SS exhibited negative selectivity, which indicates activity tracking the actual lick direction. Neurons with significant SS selectivity during the delay epoch: *n* = 19. **c**, Distinct Purkinje cell response types based on CS activity in correct trials. Left, CS activity of individual Purkinje cells within each response type. Right, population CS activity (mean ± s.e.m.) of each response type in correct trials (black) and error trials (red). ‘Lick left’ and ‘lick right’ trials were pooled. Peristimulus time histograms (PSTHs) were normalized by calculating the *z*-scored spike rate relative to the baseline spike rate before the sample epoch. Cluster 3 exhibited activity before the start of the sample epoch. This activity may be driven by the sound of the motor that repositions the pole before the start of each trial (Methods). **d**, Spatial distribution of Purkinje cells (*n* = 244 neurons) with distinct response types. Top, caudal and dorsal view of cerebellum with recorded Purkinje cells (dots). Color indicates response type identity as in **c**. Bottom, proportions of Purkinje cells within each response type in conjunction regions (*n* = 103 neurons), input-dominant regions (*n* = 74 neurons) and output-dominant regions (*n* = 67 neurons). Box-and-whisker plot shows the median and the 25th and 75th percentiles; the most extreme data points were not considered as outliers (bootstrap; Methods). Source data

**Extended Data Fig. 9:**
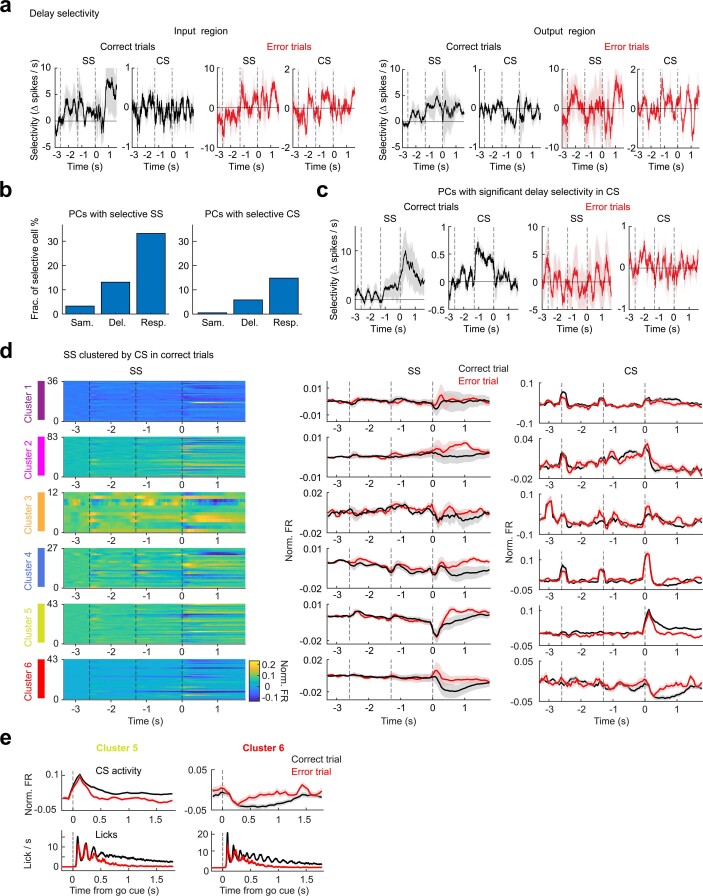
Purkinje cell simple spike and complex spike activities. **a**. Population selectivity (mean ± s.e.m. across neurons) of Purkinje cells in input-dominant regions and output-dominant regions. Selectivity of simple spikes (SS) or complex spikes (CS) in correct trials (black) and error trials (red). Purkinje cells with significant SS selectivity during the delay epoch. Same as in Fig. 7b. Trial type preference is determined by SS activity during the delay epoch in correct trials. **b**. Fraction of Purkinje cells with significant trial-type selectivity in SS or CS during specific task epochs. Left, selectivity in SS activity. Right, selectivity in CS activity. Fraction is calculated using all recorded Purkinje cells across cerebellar cortex, not limited to conjunction, input-dominant, or output-dominant regions. **c**. Population selectivity (mean ± s.e.m. across neurons) of Purkinje cells with significant selectivity in CS activity during the delay epoch. Selectivity of SS or CS in correct trials (black) and error trials (red). Trial type preference is determined by CS activity in correct trials. **d**. SS activity of different Purkinje cell populations defined by CS response profiles as in Fig. 7c. Left, SS activity of individual Purkinje cells within each population. Right, population SS activity and CS activity (mean ± s.e.m.) of each population in correct (black) and error trials (red). CS activity is the same as Fig. 7c replotted here for comparison. PSTHs were normalized by calculating the z-scored spike rate relative to the baseline spike rate before the sample epoch. **e**. CS activity differences between correct (red) and error (black) trials for cluster 5 and 6 during the response epoch are correlated with differences in licking movement. Top, CS activity from panel **d** replotted here for comparison. Bottom, lick rate in correct and error trials recorded simultaneously with the spiking activity.

We examined how preparatory activity for directional licking was encoded by Purkinje cell SS versus CS. In a subset of recordings, we could clearly distinguish Purkinje cells by the presence of CS and high-frequency SS that exhibited CS triggered suppression (Fig. 7a and Methods; 244 of 1,366 recorded neurons). We computed trial-type selectivity during the delay epoch using either SS or CS activity. We focused on the Purkinje cells from the conjunction regions as the input-dominant or output-dominant regions carried little selectivity (Extended Data Fig. 9a). In correct trials, SS exhibited robust selectivity during the delay epoch (Fig. 7b; *P* < 0.001, two-tailed *t*-test against 0). In error trials where mice licked to the direction opposite to that instructed by the sensory stimulus, SS selectivity was reversed (Fig. 7b; *P* = 0.023, two-tailed *t*-test against 0; trial type was defined by instructed lick direction and negative selectivity indicated activity that tracked actual lick direction). This indicates that SS activity during the delay epoch encoded the upcoming choice. In contrast, CS activity did not distinguish trial type in either correct or error trials (*P* = 0.162 and 0.439 respectively, two-tailed *t*-test against 0), and few Purkinje cells exhibited significant trial-type selectivity in CS activity (Extended Data Fig. 9b,c). These data indicate that information about lick direction was encoded by SS but not CS.

Despite not exhibiting trial-type selectivity, we observed diverse modulations of CS during the task, including transient activity around epoch transitions, transient activity at the beginning of the trial, buildup of activity before the motor response, or modulation during the motor response (Fig. 7c; see classification in Methods)^47–49^. The activity of all response types did not differentiate between correct and error trials (Fig. 7c and Extended Data Fig. 9d,e). Distinct CS response types were also not localized to the conjunction regions (Fig. 7d; Chi-squared test, *Χ*^2^(10) = 10.893, *P* = 0.366,). Thus, the CS modulation likely reflected processes that did not directly contribute to the maintenance of preparatory activity.

Together, these data suggest that preparatory activity is maintained by the mossy fiber pathway, with little contribution from the climbing fiber pathway.

## Discussion

**Fig. 8:**
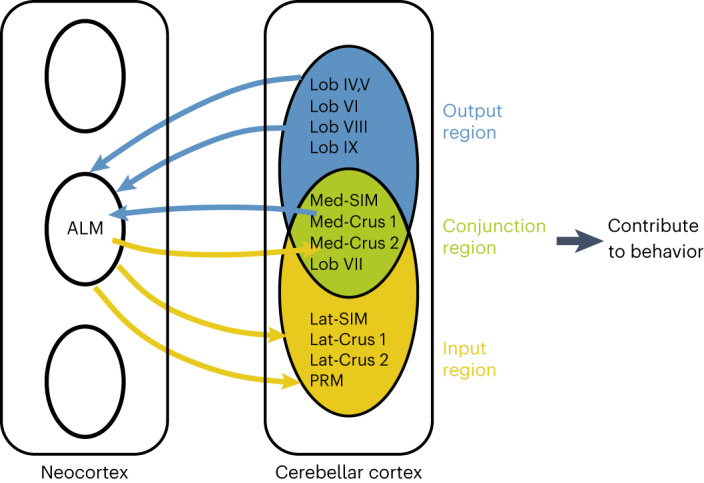
ALM cortico-cerebellar loop for motor planning of orofacial movements. The ALM exhibits divergent and convergent reciprocal connectivity with the cerebellar cortex. Conjunction regions link ALM inputs with cerebellar outputs targeting the ALM and maintain preparatory activity through reciprocal communication with the ALM. Regions outside this loop could not sustain preparatory activity and contribute little to motor planning behavior.

Our activity map analysis in relation to the cortico-cerebellar connectome revealed a cortico-cerebellar loop for motor planning of directional licking. The ALM provides diffused inputs to the cerebellar cortex, while large swaths of the cerebellar cortex send outputs back to the ALM (Fig. 1). Despite a high degree of divergence, activity supporting motor planning is selectively enriched in patches of cerebellar regions with conjunction of input–output connectivity to the ALM, covering parts of Crus 1/2, lateral simplex and vermal lobule VII (Figs. 2 and 3). Conjunction regions are required to maintain preparatory activity and motor planning behavior (Figs. 4–6). These results suggest that the cerebellar cortex functionally links the ALM inputs with outputs targeting the ALM to establish reciprocal communications that orchestrate preparatory activity (Fig. 8).

Although it is long appreciated that the neocortex and cerebellum interact during cognitive functions^1,2,4^, it remains unknown how specific neocortical and cerebellar regions organize into functional networks. Previous anatomical studies suggest closed-loop connections between corresponding regions of the neocortex and the cerebellum^3,4^. However, these studies sparsely label small groups of neurons. Other recent data suggest more divergent connectivity and complex topography^8,13–15^. Here we used transneuronal tracing from large pools of starter neurons to provide a comprehensive view of reciprocal connectivity between the ALM and the cerebellum. Notably, cerebellar regions receiving ALM inputs are largely misaligned with regions providing outputs to the ALM. For example, the strongest ALM input targets lateral simplex and lateral Crus 1/2, which do not project back to the ALM. At the same time, the identified cerebellar output regions likely send divergent projections that do not exclusively target the ALM. For example, the fastigial nucleus projects to large collections of downstream regions^15^, and its target VM thalamus also innervates large territories of motor cortex beyond the ALM^15,50^. These features of cortico-cerebellar connectivity more closely resemble open loops. Despite this divergent connectivity, preparatory activity in the cerebellum is selectively enriched in the conjunction regions (Fig. 3), and our previous mapping in neocortex found that preparatory activity is localized to the ALM^31,32^. Thus, ALM and cerebellar conjunction regions selectively form a functional network that mediates preparatory activity.

Interestingly, input and output connectivity to the ALM alone did not explain the distribution of preparatory activity in the cerebellar cortex (Fig. 3) nor participation in motor planning behavior (Fig. 4). The cerebellum is not simply upstream to the ALM because not all cerebellar regions that project to the ALM carry preparatory activity. At the same time, the cerebellum is not simply downstream to the ALM: cerebellar regions that receive ALM inputs but do not project back exhibit little preparatory activity, even though, intriguingly, the ALM provides functional inputs to both the input-dominant and conjunction regions (Extended Data Fig. 2e–i). In our analysis of the MouseLight dataset, seven of eight reconstructed pontine neurons innervate both the conjunction and input-dominant regions (Extended Data Fig. 2a–d). This suggests that diffused mossy fiber projections distribute preparatory activity to both regions. More work is needed to understand the lack of preparatory activity in the input-dominant regions and potential gating mechanisms. Purkinje cells in the conjunction regions selectively sustain preparatory activity. This indicates that reciprocal interactions between the ALM and the cerebellum, rather than each region on their own, mediate preparatory activity. Further supporting this notion, transiently breaking the cortico-cerebellar communications is sufficient to disrupt subsequent preparatory activity in both the cerebellum and ALM (Figs. 5 and 6).

A unilateral perturbation of the cerebellar conjunction regions is sufficient to disrupt preparatory activity with little recovery from other brain regions (Fig. 6). We previously found that preparatory activity is maintained in redundant modular representations across ALM hemispheres that are robust to unilateral ALM perturbations^51^. ALM hemispheres are bilaterally connected to both hemispheres of the cerebellum (Fig. 1) and unilateral cerebellar perturbations affect activity in both ALM hemispheres (Fig. 6). Thus, redundant modular organization does not appear to extend across cerebellar hemispheres. More work is still needed to understand if and how modular organization of preparatory activity maps onto the cortico-cerebellar loop.

Our study focuses on motor planning of directional licking, which requires the ALM and the fastigial nucleus^11^. Interestingly, preparatory activity is enriched in the conjunction regions defined by the fastigial output but not the dentate output (Fig. 3 and Extended Data Figs. 6 and 7). This is despite substantial overlap of ALM inputs with cerebellar regions that project to the dentate nucleus (Extended Data Fig. 7b). These findings corroborate our previous finding that the fastigial nucleus selectively mediates the planning of directional licking^11^. Other motor planning tasks appear to engage different frontal cortical regions and cerebellar nuclei^12^. We propose that the neocortex and cerebellum flexibly organize into functional networks in a task-dependent manner. Neocortical regions recruited in specific behaviors engage cerebellar regions based on their reciprocal connectivity. Given the widely divergent connectivity between the neocortex and cerebellar cortex (Fig. 1), the cerebellar cortex is in a position to combine signals from distributed neocortical areas^8^ and, in turn, influence selected cerebellar nuclei and neocortical regions. The cerebellar cortex may thus act like a railroad switch operator to flexibly establish functional networks with task-relevant neocortical regions. Regions outside the functional network contribute little to task-related activity despite input or output connectivity to the involved regions.

Whereas preparatory activity is localized to the cerebellar regions with reciprocal connectivity with the ALM, motor response-related activity is widespread (Fig. 2e; delay versus response epochs). Photostimulation of most regions of the cerebellar cortex during the response epoch biases lick direction (Fig. 4c and Extended Data Fig. 8b). Photostimulation also delays licking onset and blocks licking altogether on some trials (Extended Data Fig. 8c,d). These results are consistent with the reported role of the cerebellum in licking movement initiation^28,52^ and motor control^33,34^. Interestingly, the involved cerebellar regions appear much broader than the Crus 1/2 regions previously implicated in orofacial motor control^33,34^, and they reveal additional contributions from the posterior vermis (Extended Data Fig. 8b–d). The licking motor response likely involves cerebellar pathways beyond ALM cortico-cerebellar loop. The fastigial nucleus sends direct projections to the orofacial premotor nuclei in the medulla^15,53^. The cerebellum may contribute to licking motor control through its descending projections to the medulla. Licking initiation likely involves additional interactions between the cerebellum and other brain regions. For example, phasic signals from the midbrain are also required to trigger licking^54–56^.

A recent study delineated fastigial neurons by projection targets and molecular profiles^15^. Two distinct populations of neurons in the caudal fastigial nucleus target thalamic ventrolateral and VM nuclei, which are parts of the the ALM-projecting thalamus. The thalamic ventrolateral nuclei-projecting neurons (termed F2)^15^ receive inputs from Crus 1, and the VM-projecting population (termed F4) receive inputs from the posterior vermis. Consistent with these findings, our retrograde tracing from the ALM-projecting thalamus primarily labeled the caudal fastigial nucleus (Extended Data Fig. 3a,b). Our transneuronal retrograde tracing further identified Crus 1/2 and the posterior vermis as inputs to these thalamus-projecting fastigial neurons (Fig. 1). Both Crus 1/2 and the posterior vermis are required for motor planning of orofacial movements and enriched with preparatory activity (Figs. 3 and 4). However, neurons exhibiting preparatory activity are intermingled with neurons without selectivity in these areas. More work is needed to further resolve the detailed organization and specific functions of distinct fastigial projection neurons.

What computation might occur in the cerebellum during motor planning? Preparatory activity can be characterized as dynamics that converge to specific activity states corresponding to specific subsequent movements^16,30^. The dynamics can be described by attractor networks with two discrete attractors for ‘lick left’ and ‘lick right’^57^. The cortico-cerebellar loop may be part of this dynamical system^5,10^. One possibility is that the conjunction regions selectively amplify ALM input and pass it back to the ALM in service of maintaining choice-selective activity (that is, moving the dynamics toward one of the attractors). Alternatively, the cerebellum may play a permissive role for choice selectivity to develop. Previous analyses of preparatory dynamics reveal a nonselective ramping signal, originating outside the thalamocortical loop^29,41,57^, that permits the two attractors to develop (that is, setting up the landscape of two discrete attractors)^57^. The nonselective ramp may also encode a timing or urgency signal^25,27,58,59^. Purkinje cells may compute a ramp in the same way they compute motor timing^12,42,60^. Disambiguating the specific cerebellar computations will require simultaneous recording and perturbation of specific cerebellar signals.

## Methods

### Mice

All procedures were performed in accordance with protocols approved by the Institutional Animal Care and Use Committees at Baylor College of Medicine and the Erasmus Medical Center. This study was based on data from 66 mice (age > postnatal day (P)60, both male and female mice). Forty-one L7-cre^61^ mice crossed to Ai32 (Rosa26-LSL-ChR2-eYFP, JAX Stock 012569)^62^ mice were used for electrophysiology. Six of them were subsequently used to localize the 4 × 4 mm grid for optogenetic mapping experiments (Extended Data Fig. 8a). Four L7-cre mice crossed with Ai32 mice were used in optogenetic mapping experiments, including two mice that were also used for electrophysiology. Six L7-cre mice crossed with Ai32 mice were used in separate optogenetic experiments targeting the posterior vermis, including four mice that were also used for electrophysiology. Five L7-cre mice crossed with Ai32 mice were used in the optogenetic experiment targeting lobule IV/V, lateral simplex, medial Crus 1/2 and lobule VII in the same session (Extended Data Fig. 8e), all of which were also used for electrophysiology. Two Sim1_KJ18-cre mice^63^ crossed to Ai32 mice were used for ALM photostimulation and cerebellar recordings to measure functional connectivity. Nineteen C57BL/6J mice were used for anatomical tracing: seven mice were used to map ALM inputs to the cerebellar cortex with AAV1 virus injections in the ALM; three additional mice were used with AAV1-cre injections in ALM and secondary viruses injected in the basal pontine nuclei; five mice were used for retrograde tracing of Purkinje cells via the fastigial nucleus; and four mice were used for retrograde tracing of Purkinje cells via the dentate nucleus.

For behavioral and electrophysiological experiments, mice were individually housed in a 12:12-h reverse light:dark cycle and tested during the dark phase. Mice received all their water from daily experimental sessions (0.6 to 1.2 ml). On days not tested, mice received 0.6–1 ml of water. If mice did not maintain a stable body weight, they received supplementary water^64^. All surgical procedures were carried out aseptically under 1–2% isoflurane anesthesia. Buprenorphine Sustained Release (1 mg per kilogram body weight) and Meloxicam Sustained Release (4 mg per kilogram body weight) were used for preoperative and postoperative analgesia. A mixture of bupivacaine and lidocaine was administered topically before scalp removal. After surgery, mice were allowed to recover for at least 3 d with free access to water before water restriction.

### Surgery

Mice were prepared with a clear-skull cap and a headpost^31,64^. The scalp and periosteum over the dorsal skull were removed. A layer of cyanoacrylate adhesive (Krazy glue, Elmer) was applied to the skull. A custom headpost was placed on the skull and cemented in place with clear dental acrylic (Lang Dental Jet Repair Acrylic; 1223-clear). A thin layer of clear dental acrylic was applied over the cyanoacrylate adhesive covering the entire exposed skull, followed by a thin layer of clear nail polish (Electron Microscopy Sciences, 72180).

### Viral injection

The skull was exposed and the bregma and lambda were leveled. The glass capillary (tip opening *Φ* = 6 μm) was gently lowered, and AAV viral vectors were slowly injected in the targeted regions (injection rate <20 nl min^−1^). For ALM input tracing, 30 nl of AAV1-CAG-GFPsm-myc virus was injected in the right ALM (anterior 2.5 mm from bregma, lateral 1.5 mm, depth 0.8 mm). In additional ALM input tracing experiments, 60 nl of AAV1-CMV-Cre-GFP was injected in the right ALM, and 100 nl of AAV9-flex-tdTomato was injected in the right pontine nuclei (posterior 0.1 mm from lambda, lateral 0.5 mm, depth 5.4 mm). For monosynaptic rabies tracing, 20–30 nl of AAVretro-hSyn1-Cre-EBFP was injected in the right VM thalamus (posterior 1.4 mm from bregma, lateral 0.8 mm, depth 4.2 mm) or thalamic ventral-anterior-lateral nucleus (posterior 1.1 mm from bregma, lateral 1.0 mm, depth 3.7 mm), along with injections of helper viruses (60 nl, 1:1 mixture, AAV8-CAG-flex-oG and AAV8-CAG-FLEX-TCB-mCherry) in the left fastigial nucleus (posterior 2.7 mm from lambda, lateral 0.6 mm, depth 2.3 mm) or the left dentate nucleus (posterior 2.1 mm lambda, lateral 2.5 mm, depth 2.4 mm). Four weeks after the helper virus injection, EnvA-CMV-∆G-RV-EGFP was injected in either the left fastigial or the left dentate nucleus.

For ALM input tracing, the incubation period was 6 weeks for AAV1-GFPsm-Myc and 5 weeks for Cre-dependent tdTomato expression. For monosynaptic rabies tracing, the incubation period was 4 weeks for Cre-dependent helper viruses, and 8 d for G-deleted rabies. Afterward, mice were perfused and the brains dissected out for histology.

### Histology

For anatomical tracing experiments, mice were perfused transcardially with saline, followed by 4% paraformaldehyde/0.1 M PBS. Brains were fixed for 4 h in 4% paraformaldehyde and transferred to 10% sucrose overnight at 4 °C. Brains were then embedded in 14% gelatine and fixed in 10% formalin–30% sucrose overnight. Serial coronal sections were cut with a microtome (SM2000R, Leica) at 40 µm. The cut angle was chosen carefully to match the Allen Brain Atlas coronal sections and was kept consistent for all brains. Sections were incubated subsequently with primary antibodies at 4 °C overnight and secondary antibodies at room temperature for 2 h. For GFP staining, chicken anti-GFP primary antibody (1:2,000 dilution; Aves, GFP-1020) and Alexa Fluor 488 donkey anti-chicken secondary antibody (1:400 dilution; Jackson ImmunoResearch, 703-545-155) were used. For RFP staining, rabbit anti-RFP primary antibody (1:2,000 dilution; Rockland, 600-401-379) and Alexa Fluor 555 donkey anti-rabbit secondary antibody (1:200 dilution; Jackson ImmunoResearch, 711-165-152) were used. For myc staining, we used goat anti-myc primary antibody (1:10,000 dilution; Novus, NB600-335) and Alexa Fluor 647 donkey anti-goat (1:400 dilution; Jackson ImmunoResearch, 705-175-147). All antibodies were titrated for working solution with 2% normal horse serum–0.4% Triton–0.1 M PBS solution. For ALM mossy fiber tracing, transsynaptic labeling was visualized with DAB staining (1:150 dilution for bright-field imaging) as well as with anti-myc staining (1:10,000 dilution; for fluorescence imaging). Bright-field images were captured with Nanozoomer (2.0-RS, Hamamatsu). For fluorescence imaging, we took overviews of the brains with a ×10 objective on a fluorescence scanner (Axio Imager 2, ZEISS) or high-magnification images on a confocal microscope (LSM 700, Zeiss). Images were post-processed with ImageJ (v1.52), MATLAB R2021b and Zeiss Zen software.

For electrode localization, coronal sections at 80–100 µm were acquired in a similar manner, imaged on a fluorescence macroscope (Olympus MVX10, using software cellSens) and processed in ImageJ (v1.52).

### Behavior

The behavioral task and training have been described previously^64,65^. The stimulus was a metal pin (0.9 mm in diameter), presented at one of two possible positions (Fig. 2a). The two pole positions were 5 mm apart along the anterior–posterior axis. The posterior pole position was 5 mm from the whisker pad. A two-spout lickport (4.5 mm between spouts) was used to deliver water rewards and record licks. Behavioral data were acquired using commercial hardware and software (Bpod, Sanworks). At the start of each trial, the vertical pole was moved into reach of the whiskers (0.2 s travel time) where it remained for 1 s, after which it was retracted (retraction time 0.2 s). The sample epoch was defined as the time between the pole movement onset to 0.1 s after the pole retraction onset (1.3 s; Fig. 2a). Mice touched the object at both pole positions, typically with a different set of whiskers. The delay epoch (1.3 s) followed the sample epoch. An auditory ‘go’ cue indicated the end of the delay epoch (pure tone, 3.4 kHz, 0.1 s). Licking early during the trial was punished by a loud alarm sound (0.05 s) and a brief timeout (1–1.2 s). Licking the correct lickport after the ‘go’ cue led to a liquid reward (2–4 μl). Licking the incorrect lickport triggered a timeout (2–6 s). Trials in which mice did not lick within a 1.5-s window after the ‘go’ cue (‘ignore’) were rare and typically occurred at the end of a session. Reaction time was from the ‘go’ cue onset to the first lickport contact.

### Photostimulation

#### Cerebellar photostimulation

Light from a 473-nm laser (UltraLasers, MBL-FN-473-300mW) was controlled by an acousto-optical modulator (AOM; Quanta Tech, MTS110-A3-VIS), and focused onto the skull or brain surface (beam diameter: 400 µm at 4σ). The photostimulus had a near sinusoidal temporal profile (40 Hz) with a linear attenuation in intensity over the last 100 ms (duration: 0.4 s + 0.1 s ramp). The power values reported are the average powers. To prevent the mice from distinguishing photostimulation trials from control trials using visual cues, a ‘masking flash’ was delivered using 470-nm LEDs (Luxeon Star) near the eyes of the mice. The masking flash began as the pole started to move and continued through the end of the epoch in which photostimulation could occur.

For optogenetic mapping experiments (Fig. 4), the laser beam was aligned to lambda and a two-dimensional scanning galvo (GVSM002, Thorlabs, controlled by software WaveSurfer v1.0.2; https://www.janelia.org/) positioned the laser beam at 1 of 16 possible photostimulation locations within a 4 × 4 grid (1 mm spacing), from lambda to 3 mm posterior and 3 mm lateral. Photostimulation was performed on the left hemisphere. In each photostimulation trial, a location was chosen randomly and photostimulation was delivered during the first 500 ms of sample, delay or response epochs. Photostimulation occurred in 60% of the trials to obtain a large number of trials per condition. Photostimulation power was 1.5 or 4 mW, randomly interleaved between trials. Photostimulation was through a clear-skull cap implant^31^. The skull over the cerebellum was opaque compared to the transparent skull over the neocortex. Photostimulating locations just outside the cerebellum induced slight performance reductions (Fig. 4d), which may be caused by light scattering through the skull that affected parts of the cerebellum.

We tested the involvement of the posterior vermis in separate experiments because it was inaccessible from the dorsal surface. The laser beam was manually positioned over lobule VII through the clear-skull cap (Fig. 4). Photostimulation was delivered during the first 500 ms of sample, delay or response epochs on 40% of the trials. Photostimulation power was 1 or 3.6 mW, randomly interleaved between trials.

Photostimulation of the posterior vermis and a subset of locations within the 4 × 4 grid all induced significant effects on task performance, but these locations were tested in different experiments, making direct comparison difficult. We therefore performed an additional experiment in which we tested these regions of interest in the same session (Extended Data Fig. 8e). Using a different configuration, a scanner laser photostimulated four locations on interleaved photostimulation trials (50% of the trials): lobules IV and V, posterior 2 mm and lateral 0.5 mm from lambda (an output-dominant region); lateral simplex, posterior 0.8 mm and lateral 2 mm (an input-dominant region); medial Crus 1/2, posterior 2.5 mm and lateral 2 mm (a conjunction region); lobule VII, posterior 4.1 mm and lateral 0.5 mm (a conjunction region). Photostimulation was delivered during the first 500 ms of either the sample or the delay epoch. Photostimulation power was 1.5 mW.

For photostimulation during electrophysiology in the cerebellum (Fig. 5), the laser beam was focused on the brain surface through the recording craniotomy. Photostimulation occurred in 10% to 20% of trials during the first 500 ms of either the sample or the delay epoch. Photostimulation power ranged from 0.8 to 1.2 mW.

For photostimulation during electrophysiology in the ALM (Fig. 6), photostimulation was given through the clear-skull cap. Photostimulation was directed to the right cerebellar hemisphere and recordings were performed from both ALM hemispheres. Photostimulation occurred on 20% of trials during the first 500 ms of the delay epoch. Photostimulation power ranged from 1.0 to 1.5 mW.

#### ALM photostimulation

We photostimulated the ALM in Sim1_KJ18-cre mice crossed with Ai32 mice that expressed ChR2 in pyramidal-tract neurons innervating the pons. Photostimulation was given through the clear-skull cap (anterior 2.5 mm from bregma, lateral 1.5 mm). Photostimulation was contralateral to the recorded cerebellar hemisphere. The photostimulus was pulses of light (5-ms pulse duration) at 20 Hz (10 pulses, 455 ms) with peak powers of 15 and 40 mW and average powers of 1.5 and 4 mW. Photostimulation occurred approximately every 8 s. Photostimulation occurred on two-thirds of the trials and the other one-third of the trials did not contain photostimulation. The conditions were randomly interleaved. Mice were awake but not engaged in any task during the photostimulation. A ‘masking flash’ was delivered using 470-nm LEDs (Luxeon Star) near the eyes of the mice.

### Electrophysiology

Extracellular spikes were recorded using 64-channel Cambridge NeuroTech silicon probes (H2 acute probe, 25 μm spacing, 2 shanks). The voltage signals were amplified and digitized on an RHD2164 64-Channel Amplifier Board (Intan Technology) at 16 bit, recorded on an Intan RHD2000-Series Amplifier Evaluation System (sampling at 20,000 Hz) using Open-Source RHD2000 Interface Software from Intan Technology (version 1.5.2), and stored for offline analysis.

For cerebellum recordings, a craniotomy (diameter, 1–1.5 mm) was made over the left cerebellum. To target different cerebellar regions, craniotomies were made at various locations. The coordinates were: Lob IV and V, 2.1 mm from lambda posterior, 0.5 mm from lambda lateral; Lob VI, 3.0 mm from lambda posterior, 0.5 mm from lambda lateral ; Lob VII, VIII and IX, 4.1 mm from lambda posterior, 0.5 mm from lambda lateral; Simplex, 2.0 mm from lambda posterior, 2.0m m from lambda lateral; Crus 1, 2.5 mm from lambda posterior, 2.5 mm from lambda lateral; and Crus 2, 3.0 mm from lambda posterior, 3.0 mm from lambda lateral. A silicon probe was acutely inserted 0.7–1.5 mm below the brain surface. To minimize brain movement, a drop of silicone gel (3-4680, Dow DOWSIL) was applied over the craniotomy after the electrode was in the tissue. The tissue was allowed to settle for 10 min before the recording started. Two to six recordings were made from each craniotomy. Dil or DiR (D282, D12731, Invitrogen) was applied to the silicon probe tip to label the recording tracks (Fig. 2b,c).

For ALM recordings, two small craniotomies (diameter, 1 mm) were made over the left and right ALM (2.5 mm anterior, 1.5 mm lateral from bregma). Two silicon probes were acutely inserted 0.9–1.2 mm below the brain surface. Four to six recordings were made from each craniotomy.

All electrophysiology recordings in behaving mice were performed in well-trained expert mice (performance: 78.55% ± 6.83%, mean ± s.d. across mice). Cerebellar recordings during ALM photostimulation to map functional connectivity of the ALM to the cerebellum were performed in untrained naïve mice under awake conditions.

### Videography

Two CMOS cameras (CM3-U3-13Y3M or BFS-U3-04S2M, FLIR) were used to track orofacial movements of the mouse from side and bottom views. Videos were recorded at 200 or 294 Hz (depending on the camera model). The bottom view was acquired at 320 × 312 or 720 × 540 pixels. The side view was acquired at 370 × 340 or 400 × 480 pixels. Mice performed the task in complete darkness, and videos were acquired under IR LED illumination (940 nm).

### Anatomy data analysis

#### Alignment to CCF

We aligned each coronal section to the Allen Mouse CCF^36^ using landmark-based image registration^11^. The registration target was the 10-μm voxel CCF anatomical template. In situations where brains were cut asymmetrically, we also incorporated a projective (linear) transformation on the reference image and the raw coronal section. We manually placed control points at corresponding local landmarks in each image (Extended Data Figs. 1b and 3c). Twenty to fifty control points were placed. Next, the image was warped to the CCF using an affine transformation followed by a non-rigid transformation using b-splines^66^. Images were warped using the B-spline Grid, Image and Point based Registration package available on the MATLAB FileExchange (https://www.mathworks.com/matlabcentral/fileexchange/20057-b-spline-grid–image-and-point-based-registration/). We performed this procedure independently for each brain section.

**Extended Data Fig. 10:**
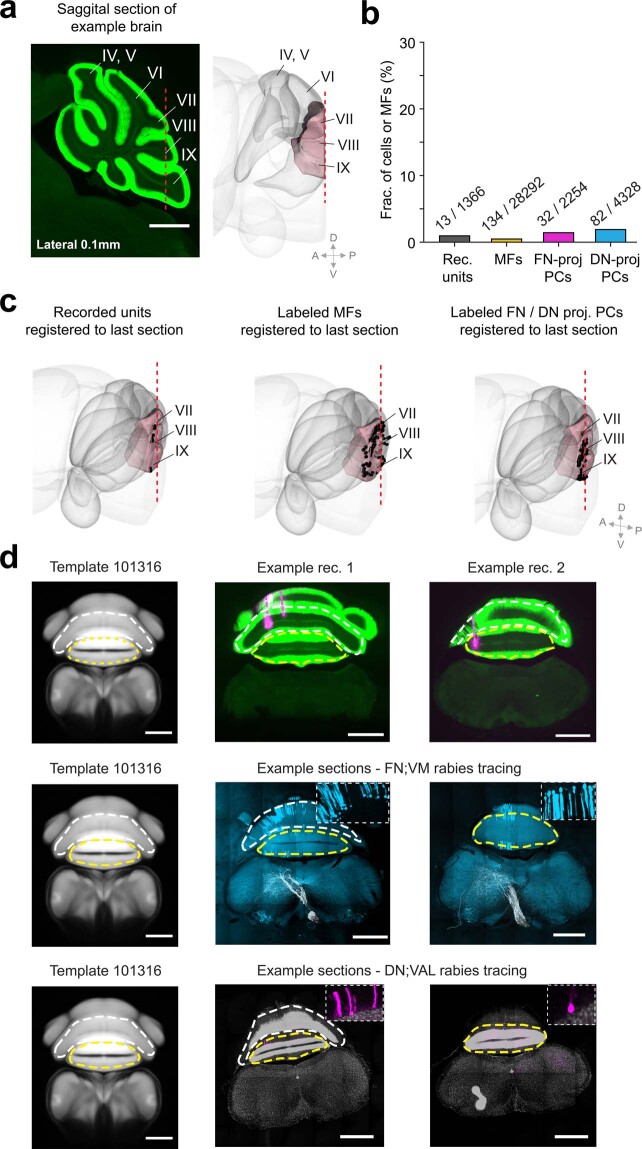
Registration of brain sections from posterior cerebellum. **a**. A small portion of the posterior cerebellum is missing in CCF anatomical template brain, which encompasses the posterior tips of vermal lobule VII, VIII, and IX. Left, sagittal view of an example brain from a L7-cre x Ai32 mouse. The red dashed line shows the cutoff. Green, eYFP expressing Purkinje cells. Right, 3D reconstruction of CCF anatomical template brain. Lobules VII, VIII and IX are highlighted. Scale bar, 1 mm. **b**. Only a small proportion of recorded units, Purkinje cells, and mossy fiber terminals were registered onto the last template section of CCF. **c**. All cells and mossy fiber terminals registered onto the last section of CCF anatomical template brain. Left, units from silicon probe recordings. Middle, labeled mossy fiber terminals. Right, retrogradely labeled Purkinje cells from either the fastigial nucleus or the dentate nucleus. **d**. Example cases of registering coronal sections from the missing posterior cerebellum onto the last section of CCF anatomical template brain. White dashed line indicates boundary of lobule VIII, yellow dashed line indicates boundary of lobule IX. Scale bars, 1 mm.

A small portion of the posterior cerebellum is missing in the CCF anatomical template brain. We found that the missing portion was small and only encompassed the most posterior tips of vermal lobules VII, VIII and IX (Extended Data Fig. 10). Any coronal sections posterior to the last section of the template brain were registered to the last template section. This encompassed a small number of mossy fiber terminals (134/28,292) and Purkinje cells (82/4,328 for retrograde labeling from the fastigial nucleus, 32/2,254 for labeling from the dentate nucleus; Extended Data Fig. 10).

#### Annotation of mossy fiber terminals and Purkinje cells

We manually annotated individual mossy fiber terminals or Purkinje cells in the raw coronal section images to obtain their [*x*, *y*] coordinates within each coronal section. We then aligned the coronal sections to the CCF, thereby obtaining their [*x*, *y*, *z*] CCF coordinates. We used the Allen Reference Atlas ontology to assign annotations to neuroanatomical regions based on their CCF coordinates. For plotting three-dimensional brain structures, we used the Allen Reference Brain (ARAv3) meshes.

#### Normalized mossy fiber projection density and Purkinje cell density

To calculate normalized mossy fiber projection density in individual lobules (Figs. 1e and 3b and Extended Data Fig. 1h,i), we first calculated the fraction of mossy fiber terminal counts in each lobule by dividing the total number of terminals across all lobules. To further compensate for lobule size, we next divided the fraction by the lobule volume. The lobule volume was calculated in CCF as the number of voxels belonging to the lobule based on Allen Reference Atlas annotations. The calculation was performed for each mouse separately to compensate for variabilities in infection rate (Extended Data Fig. 1d). In most cases (Figs. 1e and 3b and Extended Data Fig. 1i), the mossy fiber counts across both hemispheres were combined to calculate the mossy fiber density in each lobule. For Extended Data Fig. 1h, the mossy fiber density was calculated separately for each hemisphere.

Normalized Purkinje cell density (Figs. 1h and 3b and Extended Data Fig. 3j) was calculated using the same procedure as above. The Purkinje cell density was calculated for only single hemispheres because retrograde labeling from the cerebellar nucleus exclusively labeled Purkinje cells in the ipsilateral hemisphere (Fig. 1g).

For visualizations in Fig. 1e,h and Extended Data Figs. 1g and 3h, we used multivariate kernel density estimation to estimate a probability density function based on the [*x*, y, *z*] coordinates of the annotated mossy fiber terminals or Purkinje cells (kernel widths, [20 × 20 × 20 µm]). Probability density was calculated separately for each injection case, and then averaged to obtain an average density. Based on the average density, each mossy fiber terminal or Purkinje cell was assigned a color along a manually chosen color palette.

#### Definition of lobules and sub-lobules

Lobules in CCF were defined by Allen Reference Atlas annotations. Lobule simplex, Crus 1 and Crus 2 showed distinct patterns of ALM input–output connectivity within lobules (Fig. 3 and Extended Data Fig. 6). We divided them into sub-lobules based on ALM input–output connectivity via the fastigial nucleus. A threshold was set on the lateromedial axis in CCF to divide each lobule into a medial portion and a lateral portion. The thresholds were: SIM, 2.5 mm lateral from midline; Crus 1, 3.2 mm; Crus 2, 2.7 mm. In addition, we redefined cerebellar output regions via the dentate nucleus (Extended Data Fig. 7). For this analysis, lobule simplex, Crus 1, Crus 2 and the PRM were divided into sub-lobules based on ALM input–output connectivity. The thresholds were: SIM, 2.2 mm lateral from midline; Crus 1, 2.2 mm; Crus 2, 1.7 mm; PRM, 2.2 mm.

#### Definition of input-dominant, output-dominant and conjunction regions

We classified cerebellar lobules and sub-lobules into input-dominant regions, output-dominant regions or conjunction regions using a threshold (10^−11^) on the normalized mossy fiber projection density (input) and Purkinje cell density (output; Fig. 3b). Lobules and sub-lobules exceeding both the input and output thresholds were classified as conjunction regions (medial SIM, medial Crus 1, medial Crus 2 and Lob VII). Lobules and sub-lobules exceeding only the input threshold were classified as input-dominant regions (lateral SIM, lateral Crus 1, lateral Crus 2 and PRM). Lobules and sub-lobules exceeding only the output threshold were classified as output-dominant regions (COPY, Lob II–VI and VIII–X). Lobules that did not exceed either input or output threshold were excluded from analysis (Lob I, PFL and FL).

We also reclassified cerebellar lobules and sub-lobules based on their ALM input–output connectivity via the dentate nucleus using the same procedure as above (Extended Data Fig. 7). Input-dominant regions included medial SIM, medial Crus 1, medial Crus 2, medial PRM and Lob VII. Output-dominant regions included Lob IV–V, Lob IX–X, COPY, PFL and FL. Conjunction regions included lateral SIM, lateral Crus 1, lateral Crus 2 and lateral PRM. Lobules not sampled by silicon probe recordings were excluded from this analysis. In addition, Lob IV–V was excluded because silicon probe recordings did not sample the lobule tail region where the dentate-projecting Purkinje cells were located (Extended Data Fig. 7a).

We additionally tested a more continuous approach to quantify the relationship between ALM input–output connectivity and preparatory activity (Extended Data Fig. 6f,g). In CCF, we tessellated the cerebellar cortex into 100 × 100 × 100 µm voxels. The voxels evenly tiled the cerebellar cortex and could span across lobule boundaries. Within each voxel, we quantified the input connectivity strength as the number of mossy fiber terminals normalized to the total number of terminals across all voxels. We quantified the output connectivity strength as the number of Purkinje cells normalized to the total number of Purkinje cells. We quantified preparatory activity as the fraction of neurons inside the voxels carrying significant selectivity during the delay epoch, as well as their selectivity amplitude.

#### Analysis of pontine neuron reconstruction from the MouseLight database

We analyzed single pontine neuron reconstructions from the MouseLight database to examine their axonal morphology (http://ml-neuronbrowser.janelia.org/). First, we identified 21 pontine neurons using two filters: (1) soma located in the pons (query type: ‘anatomical region’; source or target locations: ‘pons’; structure: ‘soma’); (2) axon exists in the cerebellum (query type: ‘anatomical region’; source or target locations: ‘cerebellum’; structure: ‘axon’; threshold: ‘any’). Next, we defined ALM-recipient pons using anterograde tracers injected in the ALM. AAV viruses carrying fluorescent proteins were injected in the medial and lateral regions of the ALM (medial ALM: anterior 2.5 mm from bregma, lateral 1 mm; lateral ALM: anterior 2.5 mm, lateral 2 mm; 60–150 nl at a depth of 0.75 mm). AAV viruses were AAV9-syn-RFP (SignaGen, SL116027, 1.68 × 10^13^ viral genomes per ml) and pAAV-hSyn-EGFP (Addgene, 50465-AAV1, 1.1 × 10^13^ viral genomes per ml). Coronal sections containing the pons were aligned into the CCF as described above. Finally, we identified pontine neurons whose somata overlapped with ALM projections. In total, 8 pontine neurons were located in ALM-recipient pons (of 21). The distribution of pontine axons was obtained based on their CCF coordinates and Allen Reference Atlas lobule annotations (Extended Data Fig. 2).

### Behavior data analysis

#### Performance calculation and significance testing

Performance was the fraction of correct choices, excluding lick early trials and no lick trials. We also separately computed performance for ‘lick right’ and ‘lick left’ trials (Fig. 4b). Early lick rate was low in trained mice (below 10%; Extended Data Fig. 8c). Reaction time was computed across all trial types, excluding early lick trials and no lick (ignore) trials. The fractions of early lick and ignore trials were computed across all trial types (Extended Data Fig. 8c). The effects of photostimulation were quantified as the change in performance between photostimulation and control trials (Fig. 4c and Extended Data Fig. 8b,e). Change in performance was calculated separately for each photostimulation condition. In Fig. 4d and Extended Data Fig. 8f, performance change was averaged across all photostimulation spots in each region.

For each photostimulation condition, the significance of performance change was determined using bootstrap to account for variability across mice, sessions and trials (Fig. 4c and Extended Data Fig. 8b–e). We tested against the null hypothesis that the change in performance caused by photostimulation was due to normal behavioral variability. In each round of bootstrap, we replaced the original behavioral dataset with a resampled dataset in which we resampled with replacement from: (1) mice, (2) sessions performed by each mouse and (3) the trials within each session. We then computed the performance change on the resampled dataset. Repeating this procedure 1,000,000 times produced a distribution of performance changes that reflected the behavioral variability. The *P* value of the observed change in performance was computed as the fraction of times the bootstrap produced an inconsistent performance change (for example, if a performance decrease was observed during photostimulation, the *P* value was the fraction of times a performance increase was observed during bootstrap). The threshold *P* value was *α* = 0.025 (for one-tailed tests). To correct for multiple comparisons, we used the Benjamini–Hochberg procedure^67^: we first sorted the *P* values corresponding to the 16 photostimulation locations in ascending order (that is, *P*(1) ≤ *P*(2) ≤ …*P*(*i*) ≤ …≤ *P*(16)) and found the largest *i* such that *P*(*i*) ≤ $\frac{{\rm{\alpha }}\bullet i}{16}$. The performance change for grid locations, 1, …, *i*, was scored as significant.

#### Alignment of photostimulation locations to CCF

We aligned the 4 × 4 photostimulation grid into CCF. The four corners of the photostimulation grid were first marked under laser illumination used in the optogenetic experiment. Dil or DiR (D282, D12731, Invitrogen; diluted in dimethylsulfoxide, D2650, Sigma-Aldrich) was injected using pressure at each location through small craniotomies (Extended Data Fig. 8a; 50 nl, depth of 0.5 to 1 mm). The mice were perfused 2 h later and the brains were isolated for histology. Consecutive coronal sections were collected and imaged (Extended Data Fig. 8a). For each grid corner, the section with the strongest dye labeling was aligned to the CCF to yield its CCF coordinate (‘Alignment to CCF’). The remaining spots within the grid were placed into CCF by interpolation. We performed dye injections and alignments in six mice. The variability of grid locations across mice was small relative to the grid spacing (Extended Data Fig. 8a). For each grid location, we used the center location across the six mice. The photostimulation locations were then grouped into input-dominant, output-dominant or conjunction regions based on their CCF coordinates (Fig. 4d). The posterior photostimulation location outside the 4 × 4 photostimulation grid was tested using a laser positioned over lobule VII, which belonged to the conjunction regions. For comparison, the photostimulation locations were also regrouped based on ALM input–output connectivity via the dentate nucleus (Extended Data Fig. 8f).

### Electrophysiology data analysis

#### Silicon probe recording preprocessing

The extracellular recording traces were band-pass filtered (300–6 kHz). Common noise was obtained by averaging the signals across all channels and subtracting this from individual channels. Events that exceeded four standard deviations of the background were subjected to manual spike sorting.

#### Spike sorting

For cerebellar recordings, initial spike clustering was performed in principal component space^31^. The clusters were sorted manually in the data visualization tool MatClust^68^; cluster boundaries were manually adjusted. Units with a spike amplitude of less than 200 μV were generally difficult to be well isolated. The mean peak-to-peak amplitudes of the sorted units were 425 μV with a mean signal-to-noise (SNR) of 11.2.

Finally, saved clusters were evaluated by a set of manual inspections. The false alarm rate was examined by calculating the inter-spike-interval (ISI) distribution. Units with excessive ISI violations were excluded (criteria: 0.5% events with ISI < 2.5 ms; for some putative Purkinje cells with high spike rate, FA > 0.5% were accepted). Miss rate was examined by inspecting the sorted spiking events within the raw voltage traces. For each unit, a dozen randomly selected trials were visually inspected and units with excessive misses were excluded. Due to brain movements, units occasionally drifted across channels during a session. We manually inspected unit pairs recorded on adjacent channels and merged pairs that have similar spike waveforms, ISI distributions and PSTHs. In total, we obtained 1,366 single units from 36 mice across 271 sessions (Fig. 2e). Most of the recorded units were likely Purkinje cells because of their high spike rate and spike waveform shape. However, CS were not always visible in silicon probe recordings. Thus, for most recorded units, their cell types could not be inferred.

For ALM recordings, spike sorting was performed using Kilosort2 (www.github.com/MouseLand/Kilosort)^69^ followed by manually curated in Phy (https://github.com/cortex-lab/phy)^70^ and manual inspection^31^.

#### Purkinje cell spike sorting

On a subset of cerebellar recordings, CS could be detected based on their distinctive spike waveforms and low spike rate (Fig. 7a). CS always occurred on channels with SS and never on their own. In those cases, we took additional steps to isolate the Purkinje cells. First, we separately isolated SS and CS as two separate clusters using our spike sorting procedure above. Next, we manually inspected the cross-correlogram of SS and CS activity for the characteristic pause of SS activity after a CS (Fig. 7a). If the pause of SS activity was present, the SS and CS clusters were merged into a Purkinje cell unit. If the pause of SS activity was ambiguous or absent, the CS cluster was discarded and the SS cluster was kept as a regular single unit. In total, we obtained 244 clearly distinguishable Purkinje cell units of 1,366 recorded units. The mean peak-to-peak amplitude of the sorted Purkinje cell units was 559 μV with a mean SNR of 13.6. The high spike amplitude and SNR was likely due to the conservative criteria we used in their identification.

#### Registration of units to CCF

We estimated unit locations based on recording track labeling, recording depth and the lamination of activity patterns across the probe. Track location and unit locations were annotated on the raw histology images and then aligned into CCF (Fig. 2b). For penetrations located in the missing part of the anatomical template in the posterior cerebellum, we registered them to the last template section similarly to how anatomy data were processed (Extended Data Fig. 10). This encompassed a small fraction of the penetrations (14/542) and units (13/1,366). Units were assigned to individual lobules or sub-lobules based on their CCF coordinates (Extended Data Fig. 4a), and then grouped by input-dominant regions, output-dominant regions or conjunction regions.

#### Selectivity

Neurons were tested for significant trial-type selectivity using spike counts during the sample, delay or response epoch (two-tailed *t*-test, *P* < 0.01). Neurons that significantly differentiated ‘lick right’ and ‘lick left’ trials during specific epochs were deemed ‘selective’ in those epochs. Because many neurons were recorded for a limited number of error trials, we only used correct trials to quantify selectivity unless stated otherwise. To compute selectivity, we first determined the preferred trial type of each neuron using spike counts from a subset of trials (15 ‘lick right’ and ‘lick left’ trials each). Selectivity was calculated as the spike rate difference between the preferred and non-preferred trial types using the remaining trials. For Purkinje cells, the preferred trial type was calculated using SS in most cases. We also calculated selectivity using CS (Extended Data Fig. 9b,c). We separately computed selectivity using neurons with significant selectivity during sample, delay or response epochs (Fig. 2). For each epoch, the neurons’ preferred trial type was determined using spike counts within that epoch. In the text, we used the term ‘preparatory activity’ to refer to selectivity calculated during the delay epoch.

To quantify the effect of photostimulation on cerebellar selectivity, we examined all cerebellar units with significant delay epoch selectivity (Fig. 5). This included identified Purkinje cells as well as units whose cell type could not be classified. In both control and photostimulation conditions, we calculated selectivity using both correct and error trials, grouped by the instructed trial types. This is because selectivity during the delay epoch was strongly coupled to upcoming lick direction (for example, see Fig. 7b). If only correct trials were used to compute selectivity in photostimulation conditions, this would miss the trials in which photostimulation caused mice to switch future lick direction thus underestimating the effect of photostimulation on selectivity. The effect of cerebellar photostimulation on ALM selectivity was quantified in the same manner (Fig. 6).

#### Ramping activity

We classified ramping activity patterns of individual neurons using their delay activity (Extended Data Figs. 4f,g and 5g,h). Delay activity was defined as the difference in spike rate between the delay epoch and baseline (500 ms before sample epoch), calculated separately for each trial type. Ramping activity was classified using two criteria: (1) delay activity in one of the trial types was significantly different from 0 (Mann–Whitney *U* test, *P* < 0.05); (2) spike rate during the last 500 ms of the delay epoch was larger (ramping up) or smaller (ramping down) than baseline spike rate for the trial type with the largest spike rate change. Neurons with no trial-type preference or with nonsignificant delay activity were excluded from the ramping activity pattern classification^71^.

#### Clustering of Purkinje cell response types

We classified Purkinje cells into distinct response types based on the temporal profiles of their CS activity (Fig. 7). We first computed PSTHs of SS or CS in correct and error trials (‘lick left’ and ‘lick right’ trials were combined). Next, the PSTHs were normalized by calculating the *z*-scored spike rate relative to the baseline spike rate before the sample epoch. Principal component analysis was performed on PSTHs of CS in correct trials. The input to principal component analysis was an *n* × *t* matrix, where each row contained the PSTHs of individual neurons (calculated in 200-ms time bins then downsampled to 102 bins). The scores of the top six principal components were used for *k*-means clustering. We tested a range of cluster numbers (1–20), and 6 clusters produced the largest Silhouette score (Euclidean distance); therefore, we clustered Purkinje cells into six response types.

### Video data analysis

To examine whether activity related to ongoing movements contributed to neuronal trial-type selectivity (that is, preparatory activity), we used a GLM to predict neuronal activity from videos of task-performing mice. We then subtracted this movement-related activity from the neuronal firing and calculated selectivity on the residual activity (Extended Data Fig. 5).

#### Convolutional autoencoder

The videos were first compressed into a 32-dimensional embedding space using a convolutional autoencoder network (PyTorch 1.12). The network was composed of two residual blocks, each with three convolutional layers. The residual blocks were followed by two fully connected linear readout layers with output sizes of 128 and 32. We trained separate networks for each session using all trials (both correct and error). Each frame of the videos was downsampled to a size of 120 × 112 pixels for the input layer. Each network was trained for 20,000 iterations with L2 regularization.

#### Generalized linear model

We fitted a GLM for each unit to predict its responses from the videos. The predictors were the 32-dimensional embedding vectors from the convolutional autoencoder network (in 25-ms or 17-ms time steps depending on the camera model). The dependent variable was the spike rate binned at 25 ms or 17 ms (corresponding to the video frame rate). A separate GLM fitted to the spike rate shifted at five different lags (in multiples of 25-ms or 17-ms steps) in each direction. The GLM used logistic link function with Poisson spiking statistics. We used L1 regularization for the weights. The GLM parameters were fitted using 80% of the trials and tested on the remaining 20% of the trials. The variance accounted for (*R*^2^) by each unit was calculated as:

$${R}^{2}=1-\frac{\mathop{\sum }\nolimits_{i=1}^{n}{({y}_{i}-{\hat{y}}_{i})}^{2}}{\mathop{\sum }\nolimits_{i=1}^{n}{({y}_{i}-\bar{y})}^{2}}$$

Where ${y}_{i}$ is the trial-concatenated spike rate at time bin *i*, and ${\hat{y}}_{i}$ is the predicted spike rate. $n$ is the total number of time bins, and $\bar{y}$ is the mean spike rate of the unit. For each neuron, the GLM with the largest cross-validated *R*^2^ (that is, at a specific lag) was chosen for activity prediction.

To quantify the goodness of fit for activity prediction, the actual spike rate was first smoothed with a 425-ms or 340-ms bin (Extended Data Fig. 5b), and the *R*^2^ was calculated between the smoothed spike rate and predicted spike rate (Extended Data Fig. 5c). To examine if trial-type selectivity was attributable to ongoing movements, the predicted spike rate was subtracted from the actual spike rate and trial-type selectivity was calculated on the residuals (Extended Data Fig. 5d,e).

### Statistics and reproducibility

The sample sizes were similar to sample sizes used in the field: for behavior, four mice or more per condition; for electrophysiology, more than 152 ± 28 (mean ± s.e.m.) units per lobule. No statistical methods were used to determine sample size. All key results were replicated in multiple mice. Mice were randomly allocated into experimental groups. Unless stated otherwise, the investigators were not blinded to mouse group allocation during experiments and outcome assessment. Trial types were randomly determined by a computer program. During spike sorting, experimenters could not tell the trial type, so experimenters were blind to conditions. No animal data were excluded. Connectivity data from lobules that did not exceed either input or output threshold and lobules not sampled by silicon probe recordings were excluded (Lob I, II, III, PFL and FL) from analysis. Neurons with no trial-type preference or with nonsignificant delay activity were excluded from the ramping activity pattern classification. Statistical comparisons using *t*-test, bootstrap and other non-parametric tests are described above.

### Reporting summary

Further information on research design is available in the Nature Portfolio Reporting Summary linked to this article.

## Online content

Any methods, additional references, Nature Portfolio reporting summaries, source data, extended data, supplementary information, acknowledgements, peer review information; details of author contributions and competing interests; and statements of data and code availability are available at 10.1038/s41593-023-01453-x.

### Supplementary information

Reporting Summary

### Source data

Source Data Fig. 1 Source data for Fig. 1e, 1h.

Source Data Fig. 3 Source data for Fig. 3b,e–g.

Source Data Fig. 4 Source data for Fig. 4b,d.

Source Data Fig. 7 Source data for Fig. 7d.

Source Data Extended Data Fig./Table 1 Source data for Extended Data Fig. 1c,h,i.

Source Data Extended Data Fig./Table 2 Source data for Extended Data Fig. 2c,h,i.

Source Data Extended Data Fig./Table 3 Source data for Extended Data Fig. 3d,g,j.

Source Data Extended Data Fig./Table 4 Source data for Extended Data Fig. 4b.

Source Data Extended Data Fig./Table 6 Source data for Extended Data Fig. 6a.

Source Data Extended Data Fig./Table 7 Source data for Extended Data Fig. 7d.

Source Data Extended Data Fig./Table 8 Source data for Extended Data Fig. 8b–f.

## Data Availability

Data have been deposited on DANDI and can be accessed at 10.48324/dandi.000572/0.230826.0140. Source data are provided with this paper.

## Code availability

Custom codes are available from the corresponding authors upon request.
