# Supplementary material for: Activity map of a cortico-cerebellar loop underlying motor planning
Source: Nat Neurosci. 2023 Oct 9;26(11):1916–28. doi: 10.1038/s41593-023-01453-x (PMC10620095; doi:10.1038/s41593-023-01453-x)
Supplement: Supplementary file 1 — Reporting Summary [file 41593_2023_1453_MOESM1_ESM.pdf]

nature portfolio

Corresponding author(s): Nuo Li, Zhenyu Gao

Last updated by author(s): Aug 25, 2023

Reporting Summary

Nature Portfolio wishes to improve the reproducibility of the work that we publish. This form provides structure for consistency and transparency in reporting. For further information on Nature Portfolio policies, see our Editorial Policies and the Editorial Policy Checklist.

Statistics

For all statistical analyses, confirm that the following items are present in the figure legend, table legend, main text, or Methods section.

n/a	Confirmed	
☐	☒	The exact sample size (*n*) for each experimental group/condition, given as a discrete number and unit of measurement
☐	☒	A statement on whether measurements were taken from distinct samples or whether the same sample was measured repeatedly
☐	☒	The statistical test(s) used AND whether they are one- or two-sided<br>*Only common tests should be described solely by name; describe more complex techniques in the Methods section.*
☒	☐	A description of all covariates tested
☐	☒	A description of any assumptions or corrections, such as tests of normality and adjustment for multiple comparisons
☐	☒	A full description of the statistical parameters including central tendency (e.g. means) or other basic estimates (e.g. regression coefficient) AND variation (e.g. standard deviation) or associated estimates of uncertainty (e.g. confidence intervals)
☐	☒	For null hypothesis testing, the test statistic (e.g. *F*, *t*, *r*) with confidence intervals, effect sizes, degrees of freedom and *P* value noted<br>*Give P values as exact values whenever suitable.*
☒	☐	For Bayesian analysis, information on the choice of priors and Markov chain Monte Carlo settings
☒	☐	For hierarchical and complex designs, identification of the appropriate level for tests and full reporting of outcomes
☐	☒	Estimates of effect sizes (e.g. Cohen's *d*, Pearson's *r*), indicating how they were calculated

Our web collection on statistics for biologists contains articles on many of the points above.

Software and code

Policy information about availability of computer code

Data collection	Behavioral data was acquired using Bpod (Sanworks) and WaveSurfer (v 1.0.2, Janelia.org). Electrophysiological data was acquired using Intan RHD2000-Series Amplifier Evaluation System with Open-Source RHD2000 Interface Software (Intan Technology). Fluorescent images were collected with a wide-field fluorescence scanner (Axio Imager 2, ZEISS) or a confocal microscope (LSM 700, ZEISS) using software ZEN (Zeiss) and Olympus MVX10 with software cellSens (Olympus). Light microscopy images were collected with a Nanozoomer with NDP. view2 Plus(2.0-RS, Hamamatsu). Video data was collected with software FlyCapture (FLIR) and custom written programs.
Data analysis	Spike sorting was performed with MATLAB R2021b (using package UltraMegaSort2000, https://github.com/danamics/UMS2K) and Kilosort 2.0, Phy2.0 beta 1 GUI, Anaconda 3. Brain images were processed by using ImageJ (v1.52), Matlab (R2021b), Zen (Zeiss) and cellSens (Olympus) softwares. PyTorch 1.12 was used in building convolutional autoencoder network in video analysis. All analysis and statistics were performed with MATLAB R2021b using custom written codes.

For manuscripts utilizing custom algorithms or software that are central to the research but not yet described in published literature, software must be made available to editors and reviewers. We strongly encourage code deposition in a community repository (e.g. GitHub). See the Nature Portfolio guidelines for submitting code & software for further information.

Data

Policy information about availability of data

All manuscripts must include a data availability statement. This statement should provide the following information, where applicable:

- Accession codes, unique identifiers, or web links for publicly available datasets
- A description of any restrictions on data availability
- For clinical datasets or third party data, please ensure that the statement adheres to our policy

Data is available on https://doi.org/10.48324/dandi.000572/0.230826.0140
Custom codes used for analysis are available from the corresponding author upon request.

Research involving human participants, their data, or biological material

Policy information about studies with human participants or human data. See also policy information about sex, gender (identity/presentation), and sexual orientation and race, ethnicity and racism.

Reporting on sex and gender	None
Reporting on race, ethnicity, or other socially relevant groupings	None
Population characteristics	None
Recruitment	None
Ethics oversight	None

Note that full information on the approval of the study protocol must also be provided in the manuscript.

Field-specific reporting

Please select the one below that is the best fit for your research. If you are not sure, read the appropriate sections before making your selection.

☒ Life sciences ☐ Behavioural & social sciences ☐ Ecological, evolutionary & environmental sciences

For a reference copy of the document with all sections, see nature.com/documents/nr-reporting-summary-flat.pdf

Life sciences study design

All studies must disclose on these points even when the disclosure is negative.

Sample size	The sample sizes for electrophysiological and anatomical experiments were chosen to be similar to the sample sizes used in the field (Gao et al. 2018). All results were replicated in multiple subjects.
Data exclusions	No animal data was excluded. Anatomy tracing data from lobules that did not exceed either input or output threshold and lobules not sampled by silicon probe recordings was not used in activity map analysis. Neurons with no trial type preference or with non-significant delay activity were excluded from ramping activity pattern classification.
Replication	Experiments were performed using sufficient number of biological replicates (n>3 mice) to ensure reproducibility. All attempts at replication were successful.
Randomization	Animals of both sexes were randomly assigned to experimental groups. In behavioral and electrophysiological experiments, trial types and optogenetic perturbations were randomly allocated throughout the experiments.
Blinding	During experiments, trial types and optogenetic perturbations were randomly determined by computer program. During spike sorting, experimenters were blind to the trial type and conditions. Experimenters were not blinded to group allocation for neural and behavioral data analyses. All of the experiments include control conditions within the same mouse (e.g. photostimulation across different behavioral epochs; neurons responsive to photostimulation vs. those do not). Experimenters were blind to conditions during the experiment.

Reporting for specific materials, systems and methods

We require information from authors about some types of materials, experimental systems and methods used in many studies. Here, indicate whether each material, system or method listed is relevant to your study. If you are not sure if a list item applies to your research, read the appropriate section before selecting a response.

Materials & experimental systems

n/a	Involved in the study	
☐	☒	Antibodies
☒	☐	Eukaryotic cell lines
☒	☐	Palaeontology and archaeology
☐	☒	Animals and other organisms
☒	☐	Clinical data
☒	☐	Dual use research of concern
☒	☐	Plants

Methods

n/a	Involved in the study	
☒	☐	ChIP-seq
☒	☐	Flow cytometry
☒	☐	MRI-based neuroimaging

Antibodies

Antibodies used

Chicken anti-GFP primary antibody (1:2000, Aves, GFP-1020)
Rabbit anti-RFP primary antibody (1:2000, Rockland, 600-401-379)
Goat anti-myc primary antibody (1:10000, Novus, NB600-335)
Alexa fluor® 488 donkey anti-chicken secondary antibody (1:400, Jackson, 703-545-155)
Alexa fluor® 555 donkey anti-rabbit secondary antibody (1:200, Jackson, 711-165-152)
Alexa fluor® 647 donkey anti-goat secondary antibody (1:400, Jackson, 705-545-147)

Validation

Chicken anti-GFP secondary antibody: Cell Rep Methods. 2023 Feb 28;3(2):100414. doi: 10.1016/j.crmeth.2023.100414. eCollection 2023 Feb 27.
Rabbit anti-RFP secondary antibody: Cell Rep. 2022 Jun 14;39(11):110953. doi: 10.1016/j.celrep.2022.110953.
Goat anti-myc secondary antibody: Sci. Adv. 2022 Jul 15;8(28):eabn0050. doi: 10.1126/sciadv.abn0050. Epub 2022 Jul 15.
Alexa fluor® 488 donkey anti-chicken secondary antibody: Nat Commun. 2023 Jul 3;14(1):3922. doi: 10.1038/s41467-023-39496-0.
Alexa fluor® 555 donkey anti-rabbit secondary antibody: Nat Commun. 2020 Mar 13;11(1):1397. doi: 10.1038/s41467-020-15230-y
Alexa fluor® 647 donkey anti-goat secondary antibody: Nat Microbiol. 2017 Dec;2(12):1586-1591. doi: 10.1038/s41564-017-005 7-7. Epub 2017 Nov 6

Animals and other research organisms

Policy information about studies involving animals; ARRIVE guidelines recommended for reporting animal research, and Sex and Gender in Research

Laboratory animals

We used 66 mice, both male and female, aged 6-14 weeks in this study. We used 19 wild-type C57BL/6J (No. 000664), 44 L7-cre x Ai32 mice and 2 Sim1_KJ18-cre x Ai32 mice. Breeder transgenic L7-Cre (No.006207) and Ai32 (No. 012569) mice were obtained from the Jackson Labs. Transgenic Sim1_KJ18-cre mice were obtained from GENSAT.

Wild animals

This study did not use wild animals.

Reporting on sex

The experiments were done on both male and female mice.

Field-collected samples

The study did not use samples collected from the field.

Ethics oversight

All animal experiments in this study were approved in accordance with the protocols and guidelines approved by the Institutional Animal Care and Use Committees at Baylor College of Medicine and Institutional Animal Welfare Committee of the Erasmus MC.

Note that full information on the approval of the study protocol must also be provided in the manuscript.
